# *Prevotella stercorea* increases fat deposition in Jinhua pigs fed alfalfa grass-based diets

**DOI:** 10.1186/s40104-025-01217-6

**Published:** 2025-06-24

**Authors:** Qifan Zhang, Man Du, Yutian Shen, Xiaoxi Lu, Mingliang Jin, Yizhen Wang

**Affiliations:** 1 Key Laboratory of Molecular Animal Nutrition, Ministry of Education, Zhejiang University, 866 Yuhang Tang Road, Hangzhou, Zhejiang 310058 China; 2 National Engineering Research Center of Green Feeds and Healthy Livestock Industry, Zhejiang University, 866 Yuhang Tang Road, Hangzhou, Zhejiang 310058 China; 3 Key Laboratory of Animal Nutrition and Feed, Ministry of Agricultural and Rural Affairs, Zhejiang University, 866 Yuhang Tang Road, Hangzhou, Zhejiang 310058 China; 4 Zhejiang Key Laboratory of Nutrition and Breeding for High-Quality Animal Products, Zhejiang University, 866 Yuhang Tang Road, Hangzhou, Zhejiang 310058 China; 5 Present address: College of Animal Sciences, Zhejiang University, 866 Yuhang Tang Road, Hangzhou, Zhejiang 310058 China

**Keywords:** Intramuscular fat, Jinhua pig, Microbiome, *Prevotella*

## Abstract

**Background:**

Fat is a key component of body composition in both humans and animals, with intramuscular fat (IMF) being a critical determinant of pork quality. Higher IMF level enhances meat qualities such as flavor, tenderness, and juiciness, directly influencing consumer preference and market demand. Therefore, identifying microbial biomarkers associated with fat deposition is essential for improving meat quality in livestock and understanding how gut microbiota regulates host metabolism.

**Results:**

In this study, we examined changes in meat quality, fat metabolism, and gut microbiota during the pig life cycle, from weaning to marketing. We found that Jinhua pig exhibited higher IMF content and marbling score, and higher α diversity of colonic microbial communities. Microbiome Multivariate Association with Linear Models was used to identify the core genera associated with age, breed, and feed, and *Prevotella* was found to respond to both age and breed factors. The correlation analysis of fat deposition indicators with microbial genera revealed that *Prevotella* was a potential biomarker in response to IMF. In addition, the *P. stercorea *DSM 18206 (*P. stercorea*) was identified in porcine sample and administered to pseudo sterile mouse to examine the effect on IMF deposition. We found that the gavage of *P. stercorea* with alfalfa-enriched diet led to a significant increase in triglyceride (TG) and IMF contents in muscle. Metabolomic analysis further confirmed *P. stercorea* may potentially regulate fat deposition through the sphingolipid signaling pathway.

**Conclusions:**

We identified *P. stercorea* as a potential biomarker linked to higher IMF deposition and validated their role in shaping the gut microbiota and promoting fat accumulation in a mouse model, which correlated with the sphingolipid signaling pathway. These findings provide valuable insights into the role of *P. stercorea* in regulating fat deposition and metabolic health, offering implications for improving both livestock meat quality and lipid metabolism in humans.

**Supplementary Information:**

The online version contains supplementary material available at 10.1186/s40104-025-01217-6.

## Introduction

Fat is an exceptionally heterogeneous and dynamic organ for both humans and animals, playing an essential role in maintaining various physiological functions and overall health [1, 2]. In particular, intramuscular fat (IMF) plays a pivotal role in determining the quality of pork. Higher levels of IMF enhance the tenderness, flavour and juiciness of pork, which directly influences consumer preference and market demand [3, 4]. The accurate prediction of IMF content in the longissimus dorsi muscle (LDM) of pigs has become a critical aspect of improving meat quality in the livestock industry. However, while moderate fat deposition is necessary for optimal health, excessive fat accumulation can lead to several adverse health conditions, including overweight and obesity [5, 6]. These conditions are linked to a range of metabolic and cardiovascular diseases, such as type 2 diabetes and cardiovascular disease, highlighting the importance of balanced fat deposition [7–9]. A number of studies have demonstrated that gut microbes play a pivotal role in the regulation of host physiological functions, influencing energy metabolism, immune function and fat deposition and metabolism [10–12]. These microbes are intimately involved in the metabolic breakdown of carbohydrates and the overall balance of fat deposition in the body [13]. Gaining an understanding of the intricate interactions between gut microbiota, diet and fat storage is crucial for elucidating the mechanisms that influence both meat quality in livestock and metabolic health in humans.

As one of the core genera widely distributed in the human body, *Prevotella* has a wide ecological niche distribution, including the skin, oral, vagina and gastrointestinal tract in human [14–16]. Studies have shown that *Prevotella* plays a significant role in the processing of dietary components. *Prevotella* species are particularly prevalent in individuals consuming plant-based, high-fibre diets, which are characteristic of non-Western, Mediterranean, or rural African diets [17–20]. These diets, which are rich in carbohydrates and fibre, foster an environment that is conducive to the proliferation of *Prevotella*, which in turn contributes to the digestion of complex polysaccharides [21]. Recent studies have indicated the potential benefits of dietary interventions, such as the inclusion of barley kernel-based bread, which has been demonstrated to enrich *Prevotella* abundance and improve glucose metabolism [22]. Similarly, carrageenan supplementation in rats results in a marked increase in *Prevotella*, indicating that different fibre types have the potential to modulate the gut microbiome in a strain-specific manner [23]. However, despite growing evidence of role of *Prevotella* in fibre digestion, the exact mechanisms through which *Prevotella* influences host metabolism remain unclear [14, 24]. This uncertainty is partly due to the challenges in culturing *Prevotella* under strictly anaerobic conditions, as well as the significant functional diversity among different strains. While *Prevotella* is well-recognized for its association with plant-based diets, the strain-specific differences in its metabolic functions may explain the varying effects observed on host metabolism [24, 25]. Understanding these nuances is crucial for advancing our knowledge of the host-microbiome interaction, particularly the influence of gut microbes in human lipid metabolism.

In this study, we utilized a cohort of pigs from weaning to slaughter to analyze the alterations in IMF deposition and gut microbes throughout the pig life cycle. We identified *P. stercorea* as a potential biomarker associated with high IMF deposition. We further validated the role of *P. stercorea* in shaping the gut microbial community and accumulation fat deposition in mouse. Furthermore, interactions between *P. stercorea* and dietary fibre were observed, highlighting the complex interplay between gut microbiota and diet in regulating metabolic processes. These insights contribute to our understanding of how microbial communities, particularly *P. stercorea*, influence fat deposition and metabolic health.

## Materials and methods

### Animal ethics, slaughter procedure and sample collection

A 2 × 2 factorial experiment was conducted to investigate the effects of different feed types and pig breeds, at the farm of Jinhua Academy of Agricultural Sciences (Jinhua, Zhejiang, China). The study involved 60 Jinhua (JH) pigs and 60 Duroc × Landrace × Yorkshire (DLY) pigs, all born on the same day at the same farm (equal numbers of males and females). After weaning at 30 days of age, 30 JH and 30 DLY pigs were fed DLY feed (DF), a standard commercial feed, while the remaining 30 JH and 30 DLY pigs were fed JH feed (JF), an alfalfa grass-based diet with a higher crude fiber content (Table 1). The pigs were divided into four groups: JH with JF group (JJF), JH with DF group (JDF), DLY with DF group (DDF), and DLY with JF group (DJF).

**Table 1:** Ingredients and chemical composition of pig feeds (as-fed basis)

**Item**	**Jinhua pig**			**Duroc × Landrace × Yorkshire pig**		
	**28–60 d**	**61–120 d**	**121–180 d**	**28–60 d**	**61–120 d**	**121–180 d**
Ingredients, %						
Corn	60	53	50	60	63	65
Soybean meal	15	14	13	18	17	20
Fermented soybean meal	-	-	-	6	6	6
Bran	6	8	10	4	4	5
Expanded soybean	3	3	0	6	5	0
Betain	5	5	5	-	-	-
Alfalfa meal	4	10	15	-	-	-
Tea powder	3	3	3	-	-	-
Fish meal	-	-	-	2	1	0
Premix	4	4	4	4	4	4
Chemical composition						
Crude fat	3.60	3.50	3.20	5.80	5.50	5.00
Crude protein	18.50	17.30	14.50	19.00	18.20	15.00
Crude fiber	6.90	7.50	9.20	4.20	4.00	4.50
Ca	0.90	0.80	0.70	1.00	0.90	0.80
P	0.70	0.60	0.50	0.80	0.70	0.60
Digestible energy, kcal/kg	3,400.00	3,099.00	2,850.00	3,217.00	3,150.00	3,010.00

The pigs were housed in 12 pens, with 10 pigs per pen, in a barn maintained at 18–24 °C and 65%–70% humidity to ensure consistent environmental conditions. They were fed three times daily (08:00, 16:00, and 22:00) and had free access to water. At 60 d, 90 d, and 180 d of age, six pigs from each group were randomly selected, anesthetized using electroshock, and slaughtered for sampling.

All animal procedures conducted in this study were reviewed and approved by the Institutional Animal Care and Use Committee at Zhejiang University (Hangzhou, Zhejiang, China). Additionally, all procedures involving live swine, including feed, management, and slaughter, were approved by the Committee on Animal Care and Use and Committee on the Ethics of Animal Experiments of Zhejiang University (ZJU20001).

Carcass weight (CW), Loin muscle area (LMA), and three-point backfat thickness (BF) were measured, and pig liver, subcutaneous fat and visceral fat were collected and stored in liquid nitrogen for transport. The LDM was stored at −20 °C and 4 °C for subsequent molecular experiments and meat quality measurements, serum was used for physiological and biochemical indices. The digesta of the proximal colon were collected in sterile freezing tubes, snap frozen in liquid nitrogen, and stored in a refrigerator at −80 °C. Five pig colon contents were randomly selected from each group for subsequent microbiological analysis.

### Meat quality analysis

The meat qualities were measured using the LDM samples from the left side of each carcass, which were taken from the anterior end of the penultimate thoracic vertebrae of pigs about 30 cm backward. The first 10 cm of LDM samples were used to measure drip loss, the middle 10 cm for pH, meat color, and marbling score, and the posterior 10 cm for IMF and the inosine monophosphate (IMP) content.

The pH of the LDM was measured at 24 h post-mortem. The pH meter (Matthӓus pH Star, Germany) was calibrated using standard phosphate buffers at pH 4.00, 7.00, and 9.00. The glass electrode was then inserted into the center of the muscle cross-section, and the pH value was recorded once it stabilized.

Drip loss was assessed by cutting the LDM into a 2 cm × 2 cm × 2 cm cube along the muscle fibers. The initial weight was recorded. The muscle sample was then suspended in a plastic bag using a thin wire, ensuring that the meat did not touch the bag walls. The bag was sealed with a rubber band and placed in a refrigerator at 4 °C for 48 h. Afterward, the meat was removed, the residual liquid on the surface was wiped off, and the final weight was recorded. The measurement was performed in quadruplicate for each sample, and the results were averaged.

The meat color and marbling score were evaluated based on the official color and marbling quality standards (National Pork Board, Des Moines, IA, USA). The color difference value was subsequently calculated used the meat lightness, redness, and yellowness, which were measured with color difference meter (HunterLab, USA).

### Intramuscular fat and inosine monophosphate acid

The IMF content was determined using the Soxhlet extraction method. Approximately 2 g of LDM were weighed (*W*_1_), wrapped in filter paper, and dried to a constant weight (*W*_2_). The samples were then extracted with ether for 6 h, after which they were dried again to a constant weight (*W*_3_). The IMF content was calculated using the following formula:

$$X\;=\;(W_2-W_3)/W_1\;\times\;100$$

The IMP content was measured using the high-performance liquid chromatography (HPLC). Pork samples (50 mg) were added to a centrifuge tube containing 5 mL of 5% perchloric acid, then the mixture was homogenized for 10 s. After centrifugation, the samples were placed on an ice bath for 15 min. The resulting solution was transferred to a Waters tube for further analysis. The sample supernatant (20 μL) was injected onto the column (Waters Xbridge C18, USA) and quantified by HPLC (Alliance HPLC system Waters2695, USA) using UV detection (248 nm).

### Determination of triglyceride content, leptin level, and lipoprotein lipase enzyme activity

Around 1 g of LDM or liver tissue was weighed, and 9 times the volume of PBS solution was added. The samples were then homogenized, and the resulting mixture was centrifuged at 13,000 r/min for 25 min at 4 °C. The supernatant was collected for further analysis. Subsequently, the triglyceride (TG) content was determined according to the instructions of the triglyceride kits (Jiancheng Biotech, Jiangsu, China), and the absorbance values were measured at 500 nm using an enzyme meter. The leptin (LP) level and lipoprotein lipase (LPL) enzyme activity were determined using the LP and LPL Elisa kits (Meibiao Biotech Co., Ltd., Jiangsu, China).

### Quantitative real-time PCR analysis

Total RNA was extracted from LDM using Trizol reagent (Takara, Beijing, China). cDNA synthesis was performed using random primers and an RT kit (Takara, Beijing, China). Quantitative PCR was performed using SYBR Green Master Mix (Takara, Beijing, China), The primer sequences are provided in Table 2.

**Table 2:** Primer sequence of RT-PCR

Genes	Forward sequence (5′ → 3′)	Reverse sequence (5′ → 3′)
*PPARγ*	AGGACTACCAAAGTGCCATCAAA	GAGGCTTTATCCCCACAGACAC
*FABP4*	CAGGAAAGTCAAGAGCACC	ATGATACATTCCACCACCAA
*c/EBPb*	GCACAGCGACGAGTACAAGA	TATGCTGCGTCTCCAGGTTG
*ChREBP*	AGTATGTGGAGCGGAGGAAGAG	GTATTCTCGCATCACCACCTC
*ACCα*	GGTGATGGTCTATATCCCTCCTC	GATTTCTACGGTCCCTTCTGGT
*ADIPOQ*	TATGATGTCACCACTGGCAAA	TAGAGGAGCACAGAGCCAGAG
*LPL*	ACACATTCACCAGAGGGTCAC	CAATCACACGGATGGCTTCTC
*β-actin*	TCGCACTTCATGATCGAGTTG	CGACGGCCAGGTCATCAC

### Nucleic acid extraction and PCR amplification

Porcine colonic contents were collected and total bacterial genomic DNA was extracted using the FastDNA™ SPIN Kit (MP Biomedicals, USA), followed by detection by 1% agarose gel electrophoresis. Sample concentration and purity were assessed using a NanoDrop 2000 spectrophotometer (Thermo Scientific, USA) and the bacterial 16S rRNA V3-V4 hypervariable region of extracted DNA was amplified with primer pairs 338F (5'-ACTCCTACGGGAGGCAGCAG-3') and 806R (5'-GGACTACHVGGGTWTCTAAT-3') by T100 Thermal Cycler PCR thermocycler (BIO-RAD, USA).

### Bacterial 16S rRNA gene library preparation and Illumina sequencing

PCR product was purified and quantified using Qubit 4.0 (Thermo Scientific, USA), and then pooled in equimolar amounts. The library was constructed and paired-end sequenced on an Illumina Nextseq2000 platform (Illumina, San Diego, USA) according to the protocols by Majorbio Cloud Platform (Majorbio Bio-Pharm Technology Co. Ltd., Shanghai, China). The raw sequencing reads were deposited into the NCBI Sequence Read Archive (SRA) database (Accession Number: PRJNA1199807).

### Amplicon sequence processing and analysis

The PE reads obtained from sequencing were spliced according to the overlap relationship, the sequence quality was quality controlled using fastp (0.19.6) and filtered using FLASH (v1.2.11) to obtain high-quality sequences. The high-quality sequences were de-noised using the DADA2, and obtaining amplicon sequence variants (ASVs) abundance information and annotation tables. Taxonomic and annotation were performed using the Naive bayes consensus taxonomy classifier and SILVA 16S rRNA database (v138). Raw reads were deposited in the NCBI Sequence Read Archive database (accession number: PRJNA1199807, PRJNA1200223).

### Bioinformatics analysis

R-4.2.0 was utilized for bioinformatic analysis and data visualization. The genera co-correlation heatmap was generated from Spearman’s correlation coefficient and significance by “psych” package. The “gap” statistic of clustering was calculated by “clusGap” function of “cluster” R package. The bootstrap “B” parameter was set to 100, and the analysis was done with two distinct method including cluster: fanny and kmeans. The “vegan” package was used to Mantel test. Bacterial taxa differentially represented in pig breeds identified by Linear discriminant analysis Effect Size (LEfSe) online (BIC—Bioinfo Intelligent Cloud).

To identify genera associated with age, feed, and breed, we performed Microbiome Multivariate Association with Linear Models (MaAsLin2) with “MaAsLin2” package (from The Huttenhower Lab: maaslin2 – The Huttenhower Lab). Total Sum Scaling (TSS) from the “metagenomeSeq” package was used to convert absolute abundance to relative abundance (Metagenomics—CSS).

Mixed effects linear regression was performed using the “lme4” package, the model fit the effect of age growing on the normalized abundance of each genus. Prior to modelling, Cumulative Sum Scaling (CSS) was used to corrects the biases introduced by TSS with the “metagenomeSeq” package.

### Culture of *Prevotella stercorea *DSM 18206 and *Prevotella copri *DSM 18205

The standard strains were obtained from the BeNa Culture Collection (Henan, China, product numbers: BNCC351913, BNCC337399). These strains were cultured in anaerobic workstation (ELECTROTEK AW200SG, UK) at 37 ℃, filled with 80% N_2_, 10% CO_2_ and 10% H_2_. *Prevotella stercorea *DSM 18206 (*P. stercorea*) were cultured in Fastidious Anaerobe Broth (Solarbio, Beijing, China), and *Prevotella copri *DSM 18205 (*P. copri*) were cultured in modified PYG Medium (Tuopu, Shandong, China) [26].

Both strains were sequenced using Sanger method with universal primers 27F (5'-AGAGTTTGATCCTGGCTCAG-3') and 1492R (5'-GGTTACCTTGTTACGACTT-3'). The resulting sequences, in Fasta format, were compared against the rRNA/ITS databases in the NCBI Basic Local Alignment Search Tool (BLAST) before being used for gavage (Table 3).

**Table 3:** The sequences of *Prevotella* strains

Strains	Sequences (5'→ 3')
EHJ1103130-27 F	GGGCAGCTCGGCTTACACATGCAAGTCGAGGGGCAGCATGTCGGTTGCTTGCAACCGATGATGGCGACCGGCGCACGGGTGAGTAACGCGTATCCAACCTACCCTTGTCCATCGGATAACCCGTCGAAAGGCGGCCTAACACGATATGCGGTTCACAGCAGGCATCTAACGTGAACGAAATGTGAAGGAGAAGGATGGGGATGCGTCTGATTAGCTTGTTGGCGGGGTAACGGCCCACCAAGGCTACGATCAGTAGGGGTTCTGAGAGGAAGGTCCCCCACATTGGAACTGAGACACGGTCCAAACTCCTACGGGAGGCAGCAGTGAGGAATATTGGTCAATGGACGAGAGTCTGAACCAGCCAAGTAGCGTGCAGGATGACGGCCCTATGGGTTGTAAACTGCTTTTATAGGGGGATAAAGTGTGCCACGTGTGGCATATTGCAGGTACCCTATGAATAAGGACCGGCTAATTCCGTGCCAGCAGCCGCGGTAATACGGAAGGTCCGGGCGTTATCCGGATTTATTGGGTTTAAAGGGAGCGTAGGCCGTTTGGTAAGCGTGTTGTGAAATGTCGGGGCTCAACCTGGGCATTGCAGCGCGAACTGCCAGACTTGAGTGCGCAGGAAGTAGGCGGAATTCGTCGTGTAGCGGTGAAATGCTTAGATATGACGAAGAACTCCGATTGCGAAGGCAGCCTGCTGTAGCGCAACTGACGCTGAAGCTCGAAAGCGTGGGTATCGAACAGGATTAGATACCCTGGTAGTCCACGCGGTAAACGATGGATGCTCGCTGTTTGCGTCTGACGTAAGCGGCCAAGCGAAAGCGTTAAGCATCCCACCTGGGGAGTACGCCGGCAACGGTGAAACTCAAAGGATTTGACGGGGGCCCGCACAAGCGGAGGATCATGTGGTTTATTTCGATGATACGCGAGGAACCTTACCCGGGCTTGAACTGTAGGCGAACGATTCAGAGATGATGAGGCCCTTCGGGCGCCTATGGAGGTGCTGCTGGTTGCGTCAGCTCGGCCGTGAGGTGTCGGCTTAAGTGCTATACTAGCGCAACCCTTGTTCGTATGCATCGGTTGATGCGAGCACTTCTGCGAGAACTGCCTCCGTAAGTAGTGGAGGCAAGGTGGGGTATGACGTCACTATTGC
EHJ1103130-1492R	GAACGGGCCTACCCTAGGCGACCCTCGCGGTCACGGACTTTAGGCGCCCCCGGCTTTCATGGCTTGACGGGCGGTGTGTACAAGGCCCGGGAACGTATTCACCGCGCCATGGCTGATGCGCGATTACTAGCGAATCCAGCTTCGTGGGGTCGGGTTGCAGACCCCAGTCCGAACTGAGACATGTTTTAGGGATTCGAGCGTATTTGCATACACCCCGCTTTCTGTACATGCCATTGTAACACGTGTGTAGCCCCGGACGTAAGGGCCGTGCTGATTTGACGTCATCCCCACCTTCCTCACTCCTTACGGAGGCAGTCTTCGCAGAGTGCCCGGCTTCACCCGATGGCAACTACGAACAAGGGTTGCGCTCGTTATGGCACTTAAGCCGACACCTCACGGCACGAGCTGACGACAACCATGCAGCACCTCCATAGGCGCCCCGAAGGGCCTCATCATCTCTGAATCGTTCGCCTACAGTTCAAGCCCGGGTAAGGTTCCTCGCGTATCATCGAATTAAACCACATGTTCCTCCGCTTGTGCGGGCCCCCGTCAATTCCTTTGAGTTTCACCGTTGCCGGCGTACTCCCCAGGTGGGATGCTTAACGCTTTCGCTTGGCCGCTTACGTCAGACGCAAACAGCGAGCATCCATCGTTTACCGCGTGGACTACCAGGGTATCTAATCCTGTTCGATACCCACGCTTTCGAGCTTCAGCGTCAGTTGCGCTACAGCAGGCTGCCTTCGCAATCGGAGTTCTTCGTCATATCTAAGCATTTCACCGCTACACGACGAATTCCGCCTACTTCCTGCGCACTCAAGTCTGGCAGTTCGCGCTGCAATGCCCAGGTTGAGCCCCGACATTTCACAACACGCTTACCAAACGGCCTACGCTCCCTTTAAACCCAATAAATCCGGATAACGCCCGGACCTTCCGTATTACCGCGGCTGCTGGCACGGAATTAGCCGGTCCTTATTCATAGGGTACCTGCATATGCCACACGTGGCACACTTTATCCCCCTATAAAAGCAGTTTACACCCATAGGGCCGTCATCCTGCACG
EHL0404154-27 F	GGGCCAAGCTCGGCTTACACATGCAGTCGAGGGGAACGACATCGAAAGCTTGCTTTTGATGGGCGTCGACCGGCGCACGGGTGAGTAACGCGTATCCAACCTGCCCACCACTTGGGGATAACCTTGCGAAAGTAAGACTAATACCCAATGATATCTCTAGAAGACATCTGAAAGAGATTAAAGATTTATCGGTGATGGATGGGGATGCGTCTGATTAGCTTGTTGGCGGGGTAACGGCCCACCAAGGCGACGATCAGTAGGGGTTCTGAGAGGAAGGTCCCCCACATTGGAACTGAGACACGGTCCAAACTCCTACGGGAGGCAGCAGTGAGGAATATTGGTCAATGGGCGAGAGCCTGAACCAGCCAAGTAGCGTGCAGGATGACGGCCCTATGGGTTGTAAACTGCTTTTATAAGGGAATAAAGTGAGCCTCGTGAGGCTTTTTGCATGTACCTTATGAATAAGGACCGGCTAATTCCGTGCCAGCAGCCGCGGTAATACGGAAGGTCCGGGCGTTATCCGGATTTATTGGGTTTAAAGGGAGCGTAGGCCGGAGATTAAGCGTGTTGTGAAATGTAGACGCTCAACGTCTGCACTGCAGCGCGAACTGGTTTCCTTGAGTACGCACAAAGTGGGCGGAATTCGTGGTGTAGCGGTGAAATGCTTAGATATCACGAAGAACTCCGATTGCGAAGGCAGCTCACTGGAGCGCAACTGACGCTGAAGCTCGAAAGTGCGGGTATCGAACAGGATTAGATACCCTGGTAGTCCGCACGGTAAACGATGGATGCCCGCTGTTGGTCTGAACAGGTCAGCGGCCAAGCGAAAGCATTAAGCATCCCACCTGGGGAGTACGCCGGCAACGGTGAAACTCAAAGGAATTGACGGGGGCCCGCACAAGCGGAGGAACATGTGGTTTAATTCGATGATACGCGAGGAACCTTACCCGGGCTTGAATTGCAGAGGAAGGATTTGGAGACATGACGCCCTTCGGGGTCTCTGTGAAGTGCTGCATGGTTGTCGTCAGCTCGTGCCGTGAGTGTCGGCTTAGTGCATACGAGCGCACCCCTCTCTAGTGCATCAGTCAGCTGGGCACTCTGGGACACTGCCACCGTAGTGTGAGAGGTGGGGATGACGTCA
EHL0404154-1492R	CAAGAGCTTCGCTAGGCGCTCCTTACGGTCACGGACTTTAGGCGCCCCCGGCTTTCATGGCTTGACGGGCGGTGTGTACAAGGCCCGGGAACGTATTCACCGCGCCATGGCTGATGCGCGATTACTAGCGAATCCAGCTTCGTGGGGTCGGGTTGCAGACCCCAGTCCGAACTGAGACAGGCTTTAAGGATTTGATCCATTTTACATACGACCGTCTCTCTGTACCTGCCATTGTAACACGTGTGTAGCCCCGGACGTAAGGGCCGTGCTGATTTGACGTCATCCCCACCTTCCTCACACCTTACGGTGGCAGTGTCCCCAGAGTGCCCAGCTTGACCTGATGGCAACTAAGGAGAGGGGTTGCGCTCGTTATGGCACTTAAGCCGACACCTCACGGCACGAGCTGACGACAACCATGCAGCACCTTCACAGAGGCCCCGAAGGGCGTCATTGTCTCCAAATCCTTCCTCTGCAATTCAAGCCCGGGTAAGGTTCCTCGCGTATCATCGAATTAAACCACATGTTCCTCCGCTTGTGCGGGCCCCCGTCAATTCCTTTGAGTTTCACCGTTGCCGGCGTACTCCCCAGGTGGGATGCTTAATGCTTTCGCTTGGCCGCTGACCTGTTCAGACCAACAGCGGGCATCCATCGTTTACCGTGCGGACTACCAGGGTATCTAATCCTGTTCGATACCCGCACTTTCGAGCTTCAGCGTCAGTTGCGCTCCAGTGAGCTGCCTTCGCAATCGGAGTTCTTCGTGATATCTAAGCATTTCACCGCTACACCACGAATTCCGCCCACTTTGTGCGTACTCAAGGAAACCAGTTCGCGCTGCAGTGCAGACGTTGAGCGTCTACATTTCACAACACGCTTAATCTCCGGCCTACGCTCCCTTTAAACCCAATAAATCCGGATAACGCCCGGACCTTCCGTATTACCGCGGCTGCTGGCACGGAATTAGCCGGTCCTTATTCATAAGGTACATGCAAAAAGTCTCACGAGACTCACTTTATTCCCTTATAAAGCAGTTTACACCCATAGGGCGTCATCTGCACGCTACTTGGCTGGTCAGACTCTCGTCATGACATATTCTCACTGCTGCCTCCCGTAGAGTTGACCGTGTCTCAGTTCCAATGGTGGGGGACCTTT

### Mouse gavage experiment

Fresh *P. stercorea* suspensions were used for gavage, and we added a positive control group of gavaged *P. copri*, due to its known association with increased fat deposition in both pigs and mice. To explore the combined effects of fibre and *P. stercorea*, two purified mouse diets with consistent energy and protein levels were custom-designed: an alfalfa grass diet (high fibre, with 5% alfalfa grass meal added) and a control diet (no fibre), with the formulations and chemical compositions shown in Table 4.

**Table 4:** Ingredients and chemical composition of mouse feeds (as fed basis)

Item	**Control diet**	**Alfalfa diet**	**Alfalfa power**
Ingredients, %			
Casein	200.0	192.0	
L-Cystine	3.0	3.0	
Corn Starch	397.5	405.5	
Maltodextrin 10	132.0	132.0	
Sucrose	100.0	100.0	
Kaolin	50.0	0.0	
Soybean	70.0	70.0	
Alfalfa power	0.0	50.0	
Choline Bitartrate	2.5	2.5	
Vitamin Mix V10037	10.0	10.0	
Mineral Mix S10022G	35.0	35.0	
Total	1,000.0	1,000.0	
Chemical composition			
Dry matter	90.3	88.0	11.1
Crude protein	19.4	19.3	26.8
Crude fiber	0.1	1.1	30.1
Crude fat	7.9	8.2	4.5
Ash	8.0	7.9	10.5
NDF	/	/	/
ADF	/	/	/
Ca	0.6	1.0	/
P	0.3	0.3	/
Gross energy, kcal/kg	4,064.0	3,979.0	3,948.0

Thirty 5-week-old male mice (C57BL/6 J) of similar body weight (16.745 ± 0.069 g), after 3 d of pre-feeding, were randomly divided into five groups according to the diet and the strain being gavaged: alfalfa grass diet with *P. stercorea* gavaged (AS), control diet with *P. stercorea* gavaged (CS), alfalfa grass diet with *P. copri* gavaged (AP), control diet with *P. copri* (CP) and control diet with PBS gavaged (CON).

Mice were kept in a sterile environment with ad libitum access to food and water. To construct the pseudo-germ-free mouse model, the antibiotics cocktail contained 1 g/L ampicillin, 1 g/L metronidazole, 0.5 g/L neomycin, 0.5 g/L vancomycin (Yuanye Biotech Co., Ltd., Shanghai, China) was administered via oral gavage for 4 d before the microbiota transplants [27].

Fresh bacteria grown to logarithmic phase were centrifuged at 6,000 r/min for 15 min and resuspended in sterile PBS (with 0.5 g/L cysteine added) to a final concentration of 1 × 10^8^ CFU/mL [28]. Following the antibiotic treatment, 100 μL suspension was gavaged three times a week. The experiment lasted for 13 weeks, with the insulin tolerance test (ITT) and glucose tolerance test (GTT) performed during the week prior to euthanasia.

### In vivo micro-Computed Tomography (micro-CT) imaging

Mice were anesthetized with 2% isoflurane and scanned in a NEMO Micro-CT (NMC-200, Pingsheng Healthcare Inc., Shanghai, China). The following scanning parameters were used: 60 kV, 0.13 mA, 100 mm field of view, and 35 µm scan accuracy. The data sets were quantitatively analyzed and images were generated using Analyze 12.0 software. Bone, subcutaneous fat, and visceral fat were sequentially separated on consecutive sections of the CT scans, and three-dimensional (3D) volumes were calculated and used to measure the volume of the indicated adipose tissue.

### Hematoxylin-eosin staining (HE staining)

Adipose tissue was fixed in 4% paraformaldehyde for 24 h before trimming the tissue block and preparing sections. The steps were as follows: gradient dehydration (sequentially treated with 70%, 80%, 90%, 95%, and 100% ethanol for 20–30 min each time), followed by clear treatment with equal volumes of ethanol-xylene mixture and pure xylene. Next, the tissue blocks were translucently waxed by immersion in xylene-paraffin mixture and pure paraffin in turn to make wax blocks. The wax blocks were cut into thin slices and pasted on slides and dried at 45 °C. The wax blocks were then stained by HE staining. After dewaxing, HE staining was performed: hematoxylin staining for 10 min followed by washing, differentiation, dehydration, eosin staining, clearing and sealing of the sections with neutral resin. Finally, the section images were observed using a microscope and analyzed for adipocyte diameter, area and islet number using ImageProPlus and Graphpad software.

### Determination of intramuscular fat content in mouse muscle

About 20 mg of the tibialis anterior muscle or the gastrocnemius muscle was weighed, ninefold volume (w/v) of PBS solution was added and homogenized, and the supernatant was obtained by centrifugation at 13,000 r/min for 25 min at 4 ℃. The supernatant protein concentration was determined using the BCA method (P0010, Beyotime, Shanghai, China). Next, muscle IMF content was measured and calibrated with protein concentration according to the instruction manual of mouse IMF enzyme immunoassay kit (Hengyuan Biotech Co., Ltd., Shanghai, China).

### Serum biochemical parameters

The serum sample was obtained by centrifugation at 3,000 × *g* for 10 min at 4 °C, then snap-frozen in liquid nitrogen and stored at −80 °C for subsequent analysis. The serum biochemical parameters such as TG, total cholesterol (TChol), high-density lipoprotein (HDL) and low-density lipoprotein (LDL) were determined using an automated biochemical analyser (Beckman, USA) in accordance with the instructions provided.

### Determination of muscle and liver TG and TChol content

About 20 mg of the tibialis anterior muscle, gastrocnemius muscle or liver tissue was weighed, 9 times volume (w/v) of pre-cooled anhydrous ethanol was added and homogenized, and the supernatant was obtained by centrifugation at 2,500 r/min for 10 min at 4 °C. Subsequently, TG and TChol were determined according to the instructions of the TG and TChol kits (Jiancheng Biotech Co., Jiangsu, China), and the absorbance values were measured at 500 nm using an enzyme meter.

### Liquid chromatography–tandem mass spectrometry analysis (LC–MS/MS)

The solid sample (50 mg) was ground for 6 min at −10 °C and then sonicated at 5 °C for 30 min. After incubation at −20 °C for 30 min, the samples were centrifuged at 13,000 × *g* for 15 min at 4 °C. A pooled quality control (QC) sample was created by mixing equal volumes of all samples. This QC sample was processed and analyzed regularly to monitor the stability of the analysis. Samples were analyzed using a Thermo UHPLC-Q Exactive HF-X system (Thermo Scientific, USA) with an ACQUITY HSS T3 column. The mobile phases were 0.1% formic acid in water:acetonitrile (95:5) and 0.1% formic acid in acetonitrile:isopropanol:water (47.5:47.5:5). The flow rate was 0.40 mL/min, and the column temperature was set to 40 °C. The raw data were processed using Progenesis QI software to create a data matrix with sample information and metabolite intensity. Internal standard peaks and noise were removed. The data were pre-processed by retaining 80% of the features and normalizing to account for sample and instrument variability. Data from QC samples with high variability were excluded, and the data were log-transformed for further analysis.

### Glucose tolerance test

Sterile mice were placed in clean cages and fasted (water supply) for 15 h. Before the start of the experiment, mice were fasted for 12 h and then injected intraperitoneally with glucose solution according to 2 g/kg body weight, and their blood glucose was measured with a glucometer at 0, 15, 30, 60, 90 and 120 min after the injection, respectively.

### Insulin tolerance test

After 3 d of glucose tolerance test, the mice were fasted for 4 h. Insulin solution was injected intraperitoneally according to 0.75 U/kg body weight, and their blood glucose was measured with a glucometer at 0, 15, 30, 60, 90 and 120 min after the injection, respectively.

### Data plotting and statistical analysis

In this experiment, statistical analysis was performed using GraphPad Prism 8.0, SPSS software (v.26.0.0.0, SPSS Inc., Chicago, IL, USA), or R-4.2.0. The epigenetic data such as meat quality, ELISA, and relative mRNA levels were analysed using one-way analysis of variance (ANOVA) and subjected to multiple comparisons using the Turkey method. At each time point, the mean and significant differences of the four groups (JJF, JDF, DDF, and DJF) were evaluated.

To evaluate the impact of pig breed, feed type, and their interaction effects on the fat deposition indicators, a two-way ANOVA analysis was conducted in SPSS, with feed type and pig breed as fixed factors. The mean values between the two groups were obtained using independent samples *t*-test.

For the colonisation experiments using pseudo-germ-free mouse, the apparent indicators such as mouse growth, serum, and fat deposition were evaluated using one-way ANOVA, and the Turkey method was used to correct for multiple comparisons using statistical hypothesis testing. Means and significant differences between five groups (AS, AP, CS, CP and CON) were evaluated.

Analysis of microbial data was performed in R-4.2.0. Based on the ASVs information, alpha diversity indices and Bray–Curtis distance were calculated using the “vegan” package. The Permutational multivariate analysis of variance (PERMANOVA) was used to assess the percentage of variation explained by the treatment along and its statistical significance using the “vegan” package.

In MaAsLin2 linear mixed models, the TSS normalized data was used for mixed effects modelling, with day, feed type and pig breed were included as fixed effects.

CSS data were used in a mixed effects linear regression model, with age, feed type and pig breed were considered as fixed effects, while genus was considered as a random effect. Statistical significance was evaluated by one-way ANOVA or the non-parametric Kruskal–Wallis test. The *P* value was corrected with the Benjamini–Hochberg correction method.

## Results

### Differences in fat deposition phenotypes of pigs

**Fig. 1:**
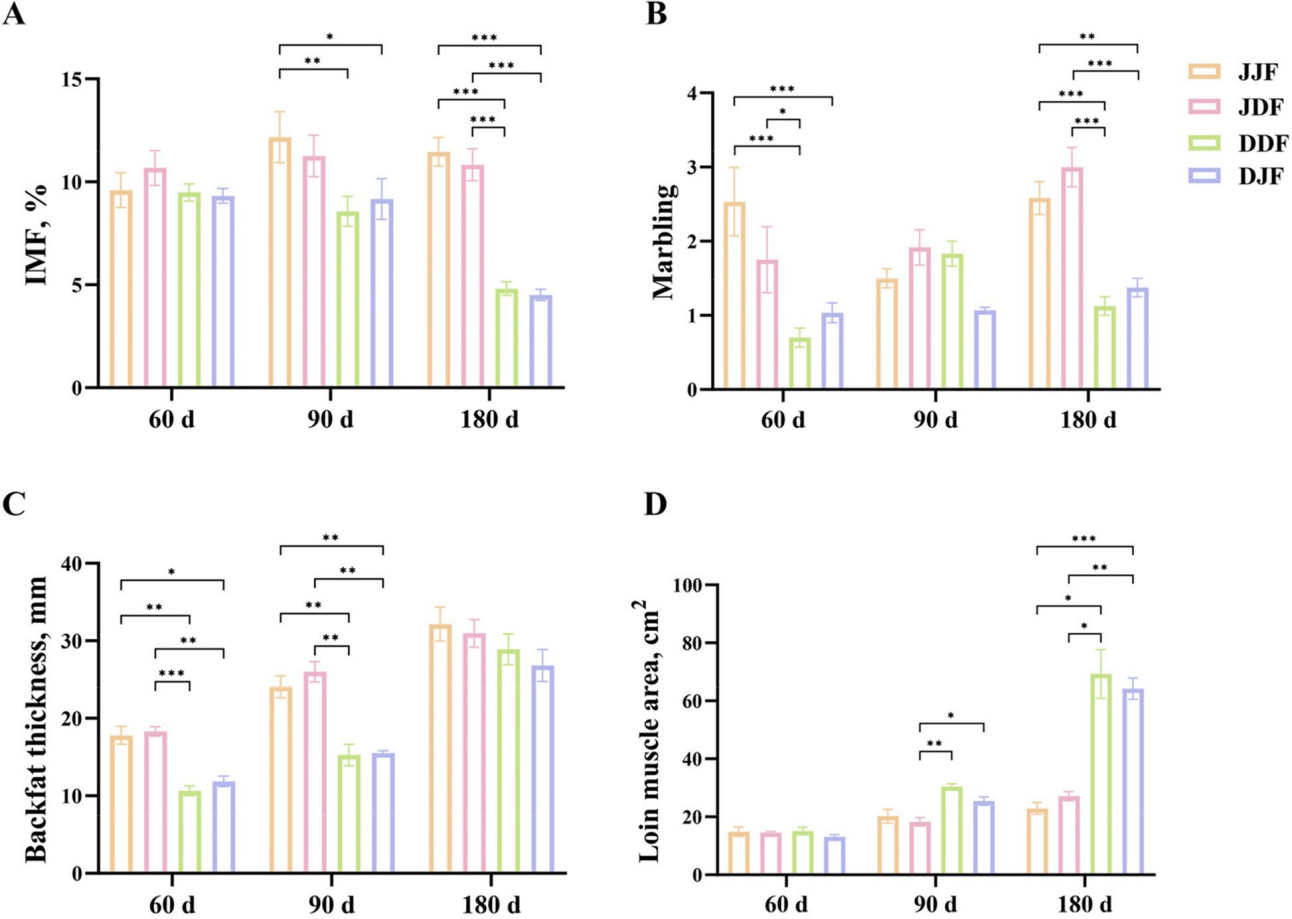
Meat quality index and carcass index related to fat deposition. **A** IMF of LDM. **B** Marbling score of LDM. **C** Backfat thickness. **D** Loin muscle area. *, ** and *** indicate *P* < 0.05, *P* < 0.01 and *P* < 0.001, respectively

In this study, we observed variation in fat deposition between different pig breeds. Specifically, JH exhibited higher levels of IMF, marbling, BF (Fig. 1A–C), and TG (Fig. S1A, Table S1) in the LDM compared to DLY when fed the same diet, at all three time points. At 180 d, the results demonstrated that the JH exhibited higher IMF content (*P* < 0.001) and marbling score (*P* < 0.01) in LDM, higher TG content in both the LDM and liver (*P* < 0.001), and lower LP level in the liver (*P *< 0.001) (Fig. S1C). Besides, the relative mRNA level of CCAAT/enhancer binding protein β (*C/EBPβ*), a regulator of adipocyte differentiation and lipid metabolism, was higher in JH compared to DLY at 90 d (*P <* 0.001; Fig. S1G). In contrast, *ADIPOQ*, a promoter of fatty acid catabolism, tended to be lower in JH at 180 d, while the difference was not statistically significant (*P* > 0.05, Fig. S1J). These results indicated that JH exhibited a superior potential for IMF deposition and fat accumulation compared to DLY, and this difference in potential was found to increase progressively with pig age.

In addition, we evaluated the effect of feed type on fat deposition, and found that there was a trend of higher TG content in LDM and liver in pigs of the same breed fed JF compared to those fed DF, but the difference was not significant (Fig. S1A, B). JH fed JF showed a higher trend in the IMF content and BF at 180 d compared to those fed DF, while the opposite trend was observed in DLY ( 0.05 < *P* < 0.1) (Fig. 1A, C). Two-way ANOVA analysis further validates the above results, revealing that pig breed was the most significant factor influencing fat deposition in this study (*P* < 0.01) while the effect of feed type was not statistically significant (*P* > 0.05) and no interaction between pig breed and feed type was observed (*P* > 0.05, Table 5).

**Table 5:** Two-way ANOVA analysis

Feed type	Pig breed	IMF	Marbling	BF	LMA
JF	JH	11.08^a^ ± 2.46	2.21^a^ ± 0.87	24.68^a^ ± 7.14	19.33^a^ ± 5.87
	DLY	7.67^b^ ± 2.72	1.16^b^ ± 0.30	18.06^b^ ± 7.17	34.25^b^ ± 23.06
DF	JH	10.92^a^ ± 2.05	2.22^a^ ± 0.95	25.09^a^ ± 6.17	19.95^a^ ± 6.17
	DLY	7.62^b^ ± 2.40	1.22^b^ ± 0.58	18.28^b^ ± 8.63	38.30^b^ ± 26.09
*P* (pig breed)		< 0.01	< 0.01	< 0.01	< 0.01
*P* (feed type)		0.86	0.82	0.86	0.58
*P* (pig breed × feed type)		0.92	0.90	0.95	0.69

All data are presented as mean ± SEM

^a,b^Values within the same column labelled with different superscript letters are significantly different

### Differences in microbial diversity

**Fig. 2:**
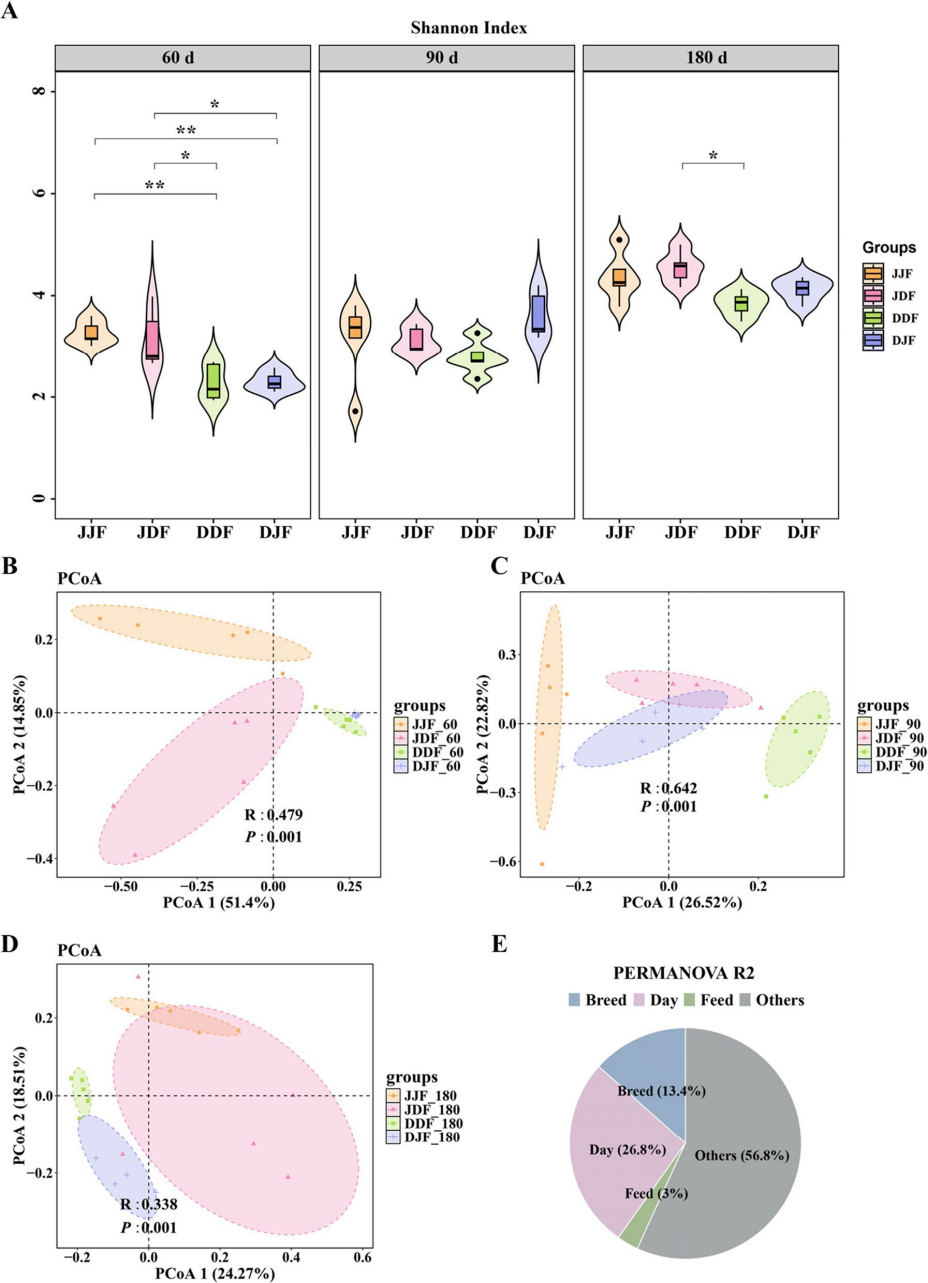
Microbial community diversity differs between age, feed type and pig breed groups. **A** The violin plots showed Shannon index of different groups among 60 d, 90 d and 180 d. **B**–**D** The PCoA for the 60 d (**B**), 90 d (**C**) and 180 d (**D**) comparison shows distinctive microbial community structure. **E** Contribution of feed type, pig breed and age to the colonic microbiota. *, ** and *** indicate *P* < 0.05, *P* < 0.01 and *P* < 0.001, respectively

The α diversity of the colonic microbiota increased from 60 to 180 d. Compared to DLY pigs, JH pigs possessed a higher Shannon Index (*P* < 0.05) (Fig. 2A) and a lower Simpson Index (*P* < 0.01) (Fig. S2C) at 60 d, indicating greater α diversity of the JH colonic microbiota. We further compared the effect of two different feed types on microbiota α diversity. At 60 d, JH fed DF showed higher α diversity, as evidenced by higher Chao 1 Index, Sobs and Ace Index (*P* < 0.05) (Fig. S2A, B, D). In contrast, DLY fed DF exhibited the opposite trend, though the differences were not statistically significant. At 180 d, the differences in α diversity attributed to feeds were no longer significant.

The Bray–Curtis distance matrix was calculated and the analysis of similarities (ANOSIM) test was performed. Principal co-ordinates analysis (PCoA) plots revealed distinct clusters in microbial community structures across different age groups (60 d, 90 d and 180 d) as well as among different feed type and pig breed groups (JJF, JDF, DDF, DJF) (Fig. 2B–D, Fig. S2E–H). To assess the influence of age, feed type and pig breed on microbial community structure, the PERMANOVA test was performed. The pie plot indicated that age (26.8%) had the largest impact on the microbial community, followed by pig breed (13.4%) (Fig. 2E).

### Identification and visualization of the core genera maturation responding to age

**Fig. 3:**
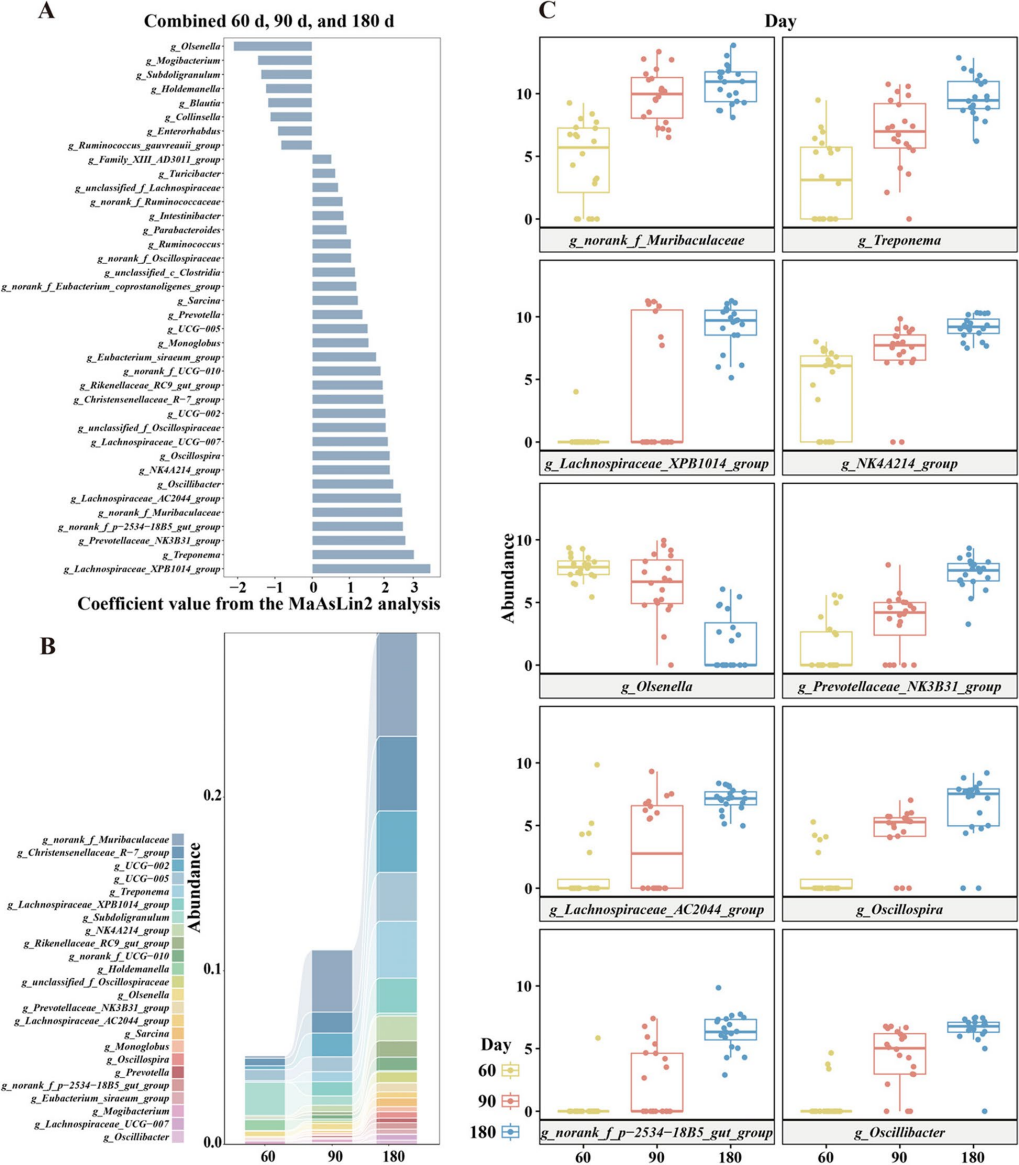
Multivariable statistical analysis and mixed-effects linear regression identified genera associated with age. **A** The coefficient values of MaAsLin2 model of top 60 genera with minimum abundance of 0.1%, used normalized relative abundance combined 60 d, 90 d and 180 d. **B** The abundance of top 24 genera associated with age were visualized by stacked plot. **C** Boxplot showed increase or decrease in abundance with age for the top ten most strongly age-associated genera

To identify the core genera associated with age, the MaAsLin2 and mixed-effects linear regression model was used. The data of all three time points were combined, then the age, pig breed and feed type were considered as “fixed_effects”. A total of 38 genera were significantly correlated (corrected *P* < 0.05) with age, of which 8 genera, including *Olsenella* (0.2%) and *Mogibacterium* (0.1%), were negatively correlated with age, and 30 genera, including *Lachnospiraceae_XPB1014_group* (0.9%) and *Treponema* (1.3%), were positively correlated with age (Fig. 3A, B), and the coefficient values and *q* values (corrected significance) were displayed (Table 6.). The absolute abundance was converted into relative abundance by TSS, and used CSS to correct the normalized data. Of the top ten genera, only *Olsenella* decreased over time, while *norank_f_Muribaculaceae* (3.2%), *Treponema* and seven other genera increased over time (Fig. 3C).

**Table 6:** The genera of MaAsLin2 model responding to age combined three time points

**Feature**	**Metadata**	**Value**	**Coefficient**	**Sstderr**	***P*-value**	***q*-value**
*Lachnospiraceae_XPB1014_group*	Day	Day	3.18	0.29	< 0.01	< 0.01
*Treponema*	Day	Day	2.74	0.30	< 0.01	< 0.01
*Prevotellaceae_NK3B31_group*	Day	Day	2.51	0.22	< 0.01	< 0.01
*norank_f_p-2534-18B5_gut_group*	Day	Day	2.45	0.23	< 0.01	< 0.01
*norank_f_Muribaculaceae*	Day	Day	2.43	0.36	< 0.01	< 0.01
*Lachnospiraceae_AC2044_group*	Day	Day	2.39	0.27	< 0.01	< 0.01
*Oscillibacter*	Day	Day	2.19	0.24	< 0.01	< 0.01
*Olsenella*	Day	Day	−2.11	0.25	< 0.01	< 0.01
*NK4A214_group*	Day	Day	2.09	0.28	< 0.01	< 0.01
*Oscillospira*	Day	Day	2.09	0.27	< 0.01	< 0.01
*Lachnospiraceae_UCG-007*	Day	Day	2.04	0.12	< 0.01	< 0.01
*unclassified_f_Oscillospiraceae*	Day	Day	1.98	0.22	< 0.01	< 0.01
*UCG-002*	Day	Day	1.98	0.28	< 0.01	< 0.01
*Christensenellaceae_R-7_group*	Day	Day	1.92	0.23	< 0.01	< 0.01
*Rikenellaceae_RC9_gut_group*	Day	Day	1.90	0.21	< 0.01	< 0.01
*norank_f_UCG-010*	Day	Day	1.85	0.22	< 0.01	< 0.01
*Eubacterium_siraeum_group*	Day	Day	1.73	0.25	< 0.01	< 0.01
*Monoglobus*	Day	Day	1.52	0.25	< 0.01	< 0.01
*UCG-005*	Day	Day	1.50	0.21	< 0.01	< 0.01
*Mogibacterium*	Day	Day	−1.46	0.23	< 0.01	< 0.01
*Subdoligranulum*	Day	Day	−1.38	0.25	< 0.01	< 0.01
*Prevotella*	Day	Day	1.36	0.21	< 0.01	< 0.01
*Holdemanella*	Day	Day	−1.25	0.24	< 0.01	< 0.01
*Sarcina*	Day	Day	1.24	0.23	< 0.01	< 0.01
*norank_f_Eubacterium_coprostanoligenes_group*	Day	Day	1.20	0.17	< 0.01	< 0.01
*Blautia*	Day	Day	−1.19	0.24	< 0.01	< 0.01
*unclassified_c__Clostridia*	Day	Day	1.16	0.16	< 0.01	< 0.01
*Collinsella*	Day	Day	−1.13	0.22	< 0.01	< 0.01
*norank_f_Oscillospiraceae*	Day	Day	1.05	0.28	< 0.01	< 0.01
*Ruminococcus*	Day	Day	1.05	0.22	< 0.01	< 0.01
*Parabacteroides*	Day	Day	0.93	0.28	< 0.01	0.01
*Enterorhabdus*	Day	Day	−0.93	0.20	< 0.01	< 0.01
*Intestinibacter*	Day	Day	0.85	0.32	0.01	0.03
*Ruminococcus_gauvreauii_group*	Day	Day	−0.84	0.26	< 0.01	0.01
*norank_f_Ruminococcaceae*	Day	Day	0.83	0.23	< 0.01	< 0.01
*unclassified_f_Lachnospiraceae*	Day	Day	0.70	0.14	< 0.01	< 0.01
*Turicibacter*	Day	Day	0.63	0.23	0.01	0.03
*Family_XIII_AD3011_group*	Day	Day	0.53	0.13	< 0.01	< 0.01

The “lme4” R package was performed to construct a mixed effects linear regression model for the normalized abundance (TSS + CSS) of genera in response to increasing age. Similarly, 8 genera including *Olsenella* and *Subdoligranulum* (0.9%), were negatively correlated with age, while *Lachnospiraceae_XPB1014_group* showed the strongest positive correlation. Notably, the *Lachnospiraceae_UCG-007* (0.1%) was also included in the positive correlating genera with age (Fig. S3A, D).

### Identification of the genera responding to pig breed and feed type

**Fig. 4:**
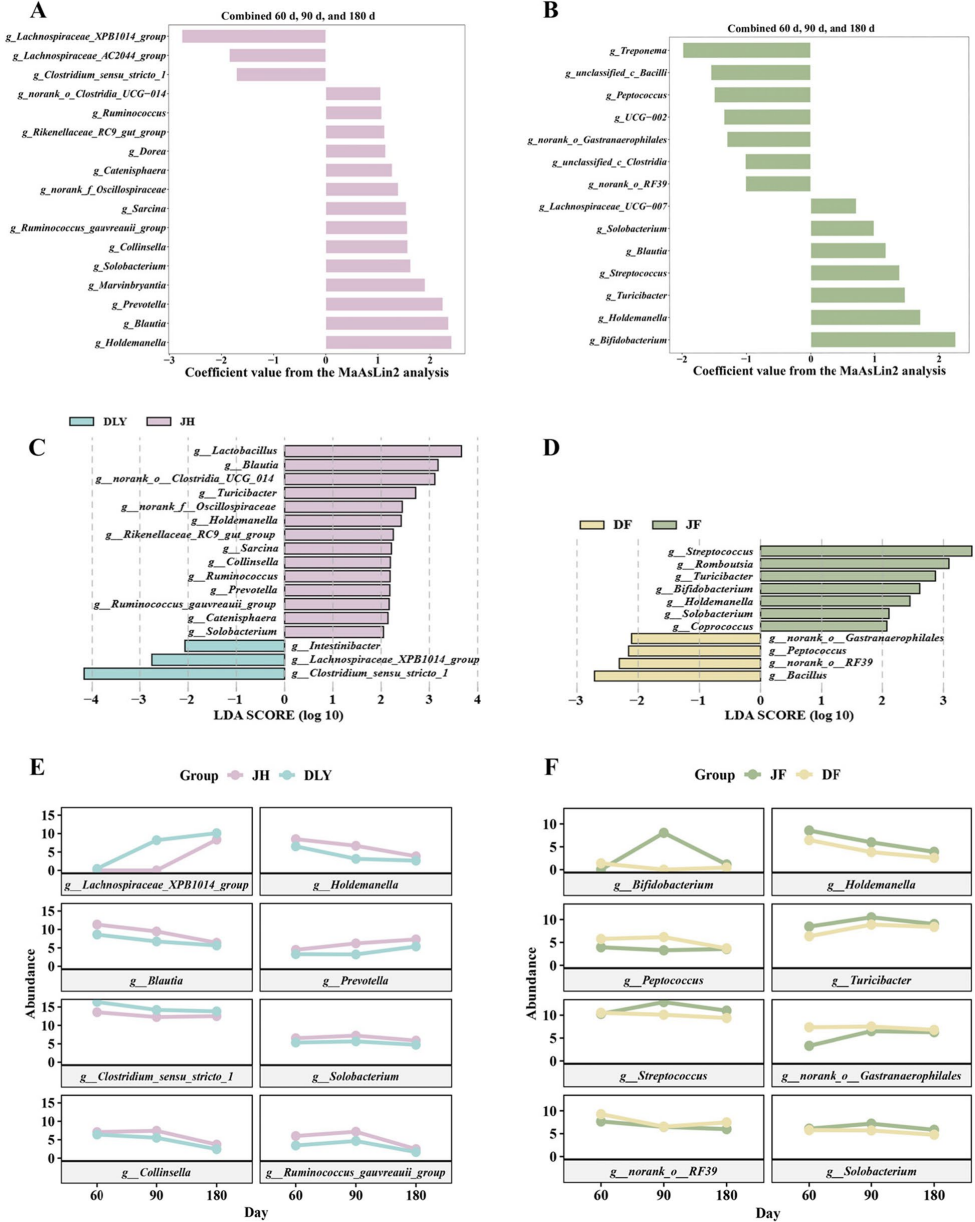
Multivariable statistical analysis and LEfSe analysis identified genera associated with feed type and pig breed. **A** The coefficient values of MaAsLin2 model responding to breed factor with normalized relative abundance in combined all time points dataset. **B** The coefficient values of MaAsLin2 model responding to feed factor in combined all time points dataset. **C**, **D** LEfSe analysis revealed the most differentially abundant taxa with LDA threshold > 2.0 between pig breeds (**C**) and between feed types (**D**). **E**, **F** Lineplots showed abundance with age of the strongly breed-associated genera (**E**) and feed- associated genera (**F**) identity by MaAsLin2 and LEfSe analysis

In combined time dataset, three genera had a significant correlation with DLY, including *Lachnospiraceae_XPB1014_group*, *Lachnospiraceae_AC2044_group*, and *Clostridium_sensu_stricto_1*, and 14 genera strongly correlated with JH, including *Holdemanella*, *Blautia*, and *Prevotella* (Fig. 4A, Table 7)*.* In the separate day dataset analysis, the association of *Holdemanella* and *Blautia* was disappeared at 180 d, while the *Prevotella* and *Solobacterium* showed a correlation with JH starting after 90 d (Fig. S3F, G, Table S2–S4).

**Table 7:** The genera of MaAsLin2 model responding to pig breed combined three time points

Feature	Metadata	Value	Coefficient	Sstderr	*P*-value	*q*-value
*Lachnospiraceae_XPB1014_group*	Breed	JH	−2.76	0.58	< 0.01	< 0.01
*Holdemanella*	Breed	JH	2.41	0.48	< 0.01	< 0.01
*Blautia*	Breed	JH	2.35	0.47	< 0.01	< 0.01
*Prevotella*	Breed	JH	2.25	0.43	< 0.01	< 0.01
*Marvinbryantia*	Breed	JH	1.90	0.60	< 0.01	0.01
*Lachnospiraceae_AC2044_group*	Breed	JH	−1.85	0.54	< 0.01	0.01
*Clostridium_sensu_stricto_1*	Breed	JH	−1.71	0.38	< 0.01	< 0.01
*Solobacterium*	Breed	JH	1.63	0.34	< 0.01	< 0.01
*Collinsella*	Breed	JH	1.57	0.43	< 0.01	< 0.01
*Ruminococcus_gauvreauii_group*	Breed	JH	1.56	0.52	< 0.01	0.01
*Sarcina*	Breed	JH	1.54	0.46	< 0.01	0.01
*norank_f_Oscillospiraceae*	Breed	JH	1.39	0.55	0.01	0.04
*Catenisphaera*	Breed	JH	1.27	0.42	< 0.01	0.01
*Dorea*	Breed	JH	1.15	0.43	0.01	0.03
*Rikenellaceae_RC9_gut_group*	Breed	JH	1.13	0.41	0.01	0.03
*Ruminococcus*	Breed	JH	1.07	0.43	0.02	0.04
*norank_o__Clostridia_UCG-014*	Breed	JH	1.05	0.33	< 0.01	0.01

Top ten genera ranked by coefficient values were considered as potential candidate genera responding to breed, and eight of them showed the same enrichment in LEfSe analysis (Fig. 4C). Among these, *Prevotella* and *Lachnospiraceae_XPB1014_group* were increased over growth (Fig. 4E).

Regarding feed type, 8 genera were identified both by MaAsLin2 and LEfSe analysis in combined time dataset, *Peptococcus*, *norank_o_Gastranaerophilales,* and *norank_o_RF39* were associated with DF, while *Bifidobacterium*, *Holdemanella* and three other genera were associated with JF (higher crude fibre content) (Fig. 4B, D, Table 8). The analysis of separate day datasets revealed 90 d showed the strongest response to feed type, with *Peptococcus* showed the connection with DF. And the JF connected genera, including *Bifidobacterium*, *Streptococcus*, *Turicibacter* and *Solobacterium*, exhibited the highest abundance at 90 d (Fig. 4F, Fig. S3C, Table S5–S7).

**Table 8:** The genera of MaAsLin2 model responding tofeed type combined three time points

Feature	Metadata	Value	Coefficient	Sstderr	*P*-value	*q*-value
*Bifidobacterium*	Feed	JF	2.26	0.77	0.01	0.02
*Treponema*	Feed	JF	−1.98	0.60	< 0.01	0.01
*Holdemanella*	Feed	JF	1.71	0.48	< 0.01	< 0.01
*unclassified_c__Bacilli*	Feed	JF	−1.55	0.61	0.01	0.04
*Peptococcus*	Feed	JF	−1.50	0.60	0.02	0.04
*Turicibacter*	Feed	JF	1.47	0.46	< 0.01	0.01
*Streptococcus*	Feed	JF	1.39	0.45	< 0.01	0.01
*UCG-002*	Feed	JF	−1.35	0.55	0.02	0.05
*norank_o__Gastranaerophilales*	Feed	JF	−1.30	0.45	0.01	0.02
*Blautia*	Feed	JF	1.18	0.47	0.02	0.04
*unclassified_c__Clostridia*	Feed	JF	−1.02	0.32	< 0.01	0.01
*norank_o__RF39*	Feed	JF	−1.01	0.41	0.02	0.05
*Solobacterium*	Feed	JF	0.99	0.34	0.01	0.02
*Lachnospiraceae_UCG-007*	Feed	JF	0.72	0.23	< 0.01	0.01

### Identification of potentional microbe biomarker for high IMF

IMF development in LDM is influenced by multiple factors, including genetics, environment and age [29]. Studies has confirmed an inevitable increase in the concentration of fat in muscle in animal’s later life [30], and the IMF content rising progressively with age in pigs [31, 32]. In this research, we analyzed meat quality indexes, carcass indexes, and other indexes related to IMF development and fat deposition at three time points. As indicated above, JH exhibited higher IMF and TG content in LDM, therefore we considered 180 d located in the maturation stage of fat deposition for subsequent microbial analyses.

**Fig. 5:**
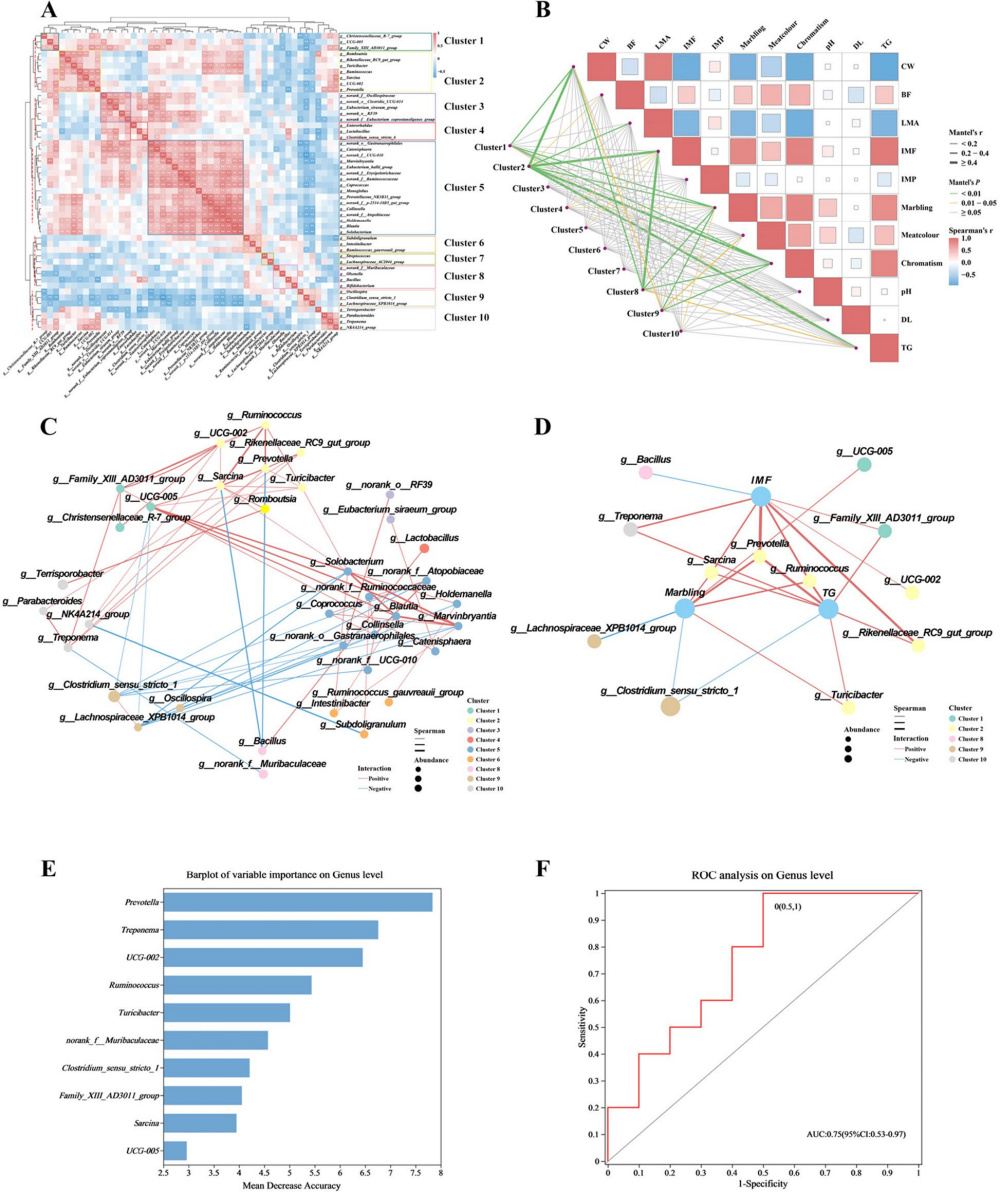
Correlation analysis and random forest model identified biomarker indicate high IMF. **A** Heatmap of the co-correlation of top 50 genera, the TSS abundance was calculated for the Pearson’s rank correlation coefficient, 10 cluster were generated (marked by different colored boxes). **B** The 10 most clusters were related to meat qualities and carcass indicators by Mantel tests using Spearman's correlation analysis. **C** Correlation network generated by the top 50 genera. **D** Correlation network between top 50 genera and indicators related to IMF deposition. **E** Random forest model was constructed by all samples at 180 d, the number of decision tree was set to 500. **F** The receiver operating characteristic curve of the random forest model

We selected top 50 genera for correlation analysis across all samples, and the abundance at 180 d was calculated to generate a heatmap of the Pearson’s rank correlation coefficient (Fig. 5A). Ten clusters were illustrated in the dendrogram on the left side of heat map (marked by the red dashed line), and the goodness of clustering was further confirmed by “clusGap” function of “cluster” R package (Fig. S4A, B). The (highlighted in yellow box) consisted of seven genera, including *Prevotella, Ruminococcus* and other fibre-degrading genera. Next, we performed Mantel test between the ten clusters and carcass and meat quality indicators (Fig. 5B, Table S8). Cluster 2 showed the strongest association with IMF (R = 0.65) and TG (R = 0.56), indicating a significant association between Cluster 2 and IMF deposition.

Correlation network generated by the top 50 genera, genera of Cluster 2 showed positive correlations with genera of Cluster 1, Cluster 10, and *Solobacterium* of Cluster 5, negative correlations with *Bacillus* of Cluster 8 (Fig. 5C). Further, we found *Prevotella, Sarcina* and *Ruminococcus* were positively correlated with IMF, Marbling and TG, and *Prevotella* showed the strongest connection to IMF (Coefficient = 0.89) (Fig. 5D).

To validate the positive correlation between *Prevotella* and high IMF deposition, the top 50 genera were used to construct a random forest model. All samples at180 d were categorized into high and low IMF groups based on IMF content (*n* = 10) (Fig. 5E). The ROC of the model was plotted and the AUC was 0.75, which proved the model was highly accurate (Fig. 5F). The bar graph demonstrated *Prevotella* showed the highest impact on model accuracy. Notably, four of the top five important genera belonged to Cluster 2, except for *Treponema*, which from Cluster 10. These results strongly suggest that *Prevotella* is strongly associated with IMF deposition, making it a potential microbial biomarker of response to high IMF content.

### Selection and cultivation of potentially functional strains

To identify function strains responding to high IMF deposition in pig samples, all ASVs belonging to the *Prevotella* were selected and the sequences in Fasta format were subjected to sequence comparison in the NCBI Basic Local Alignment Search Tool of the rRNA/ITS databases. A total of 12 ASVs belonged to *Prevotella,* based on the comparison results, 4 ASVs were identified as *P. stercorea* and possessed the highest relative abundance in the pig samples (Table S9, S10). Therefore, we purchased the standard strain *P. stercorea* and cultured under anaerobic conditions.

### Gavage in pseudo-germ-free mouse verified the effect of *P. stercorea* in vivo

**Fig. 6:**
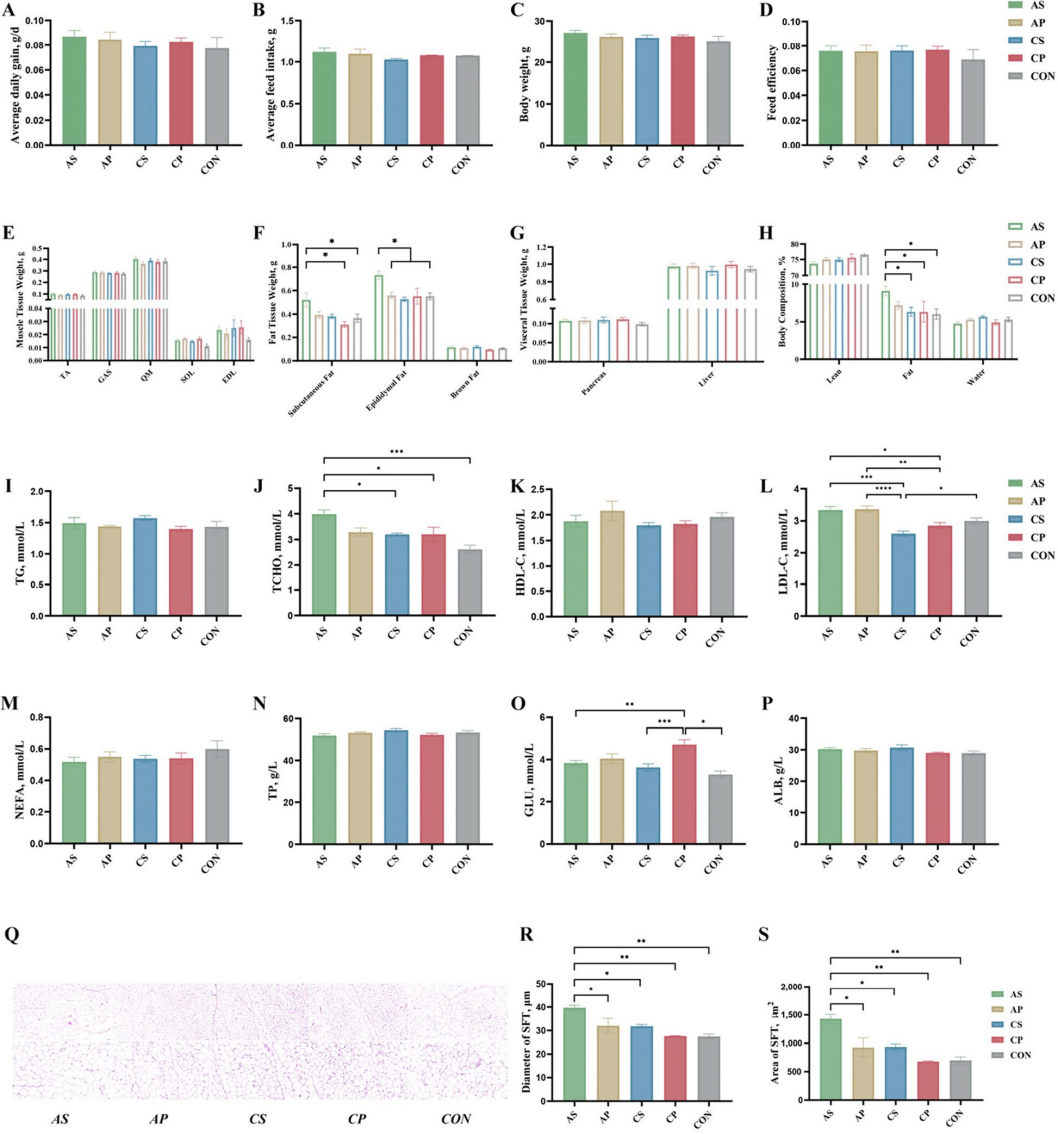
The influence of *P. stercorea* on growth factors, serum components, and adipocyte development in mice. **A**–**D** The body weight and feed intake of mice. **E**–**H** The tissue weight and body composition. **I**–**P** The serum parameters. **Q** Histological images depict mouse subcutaneous adipose tissue using haematoxylin and eosin staining. **R**–**S** The average diameter and average area of mouse subcutaneous adipose. *, ** and *** indicate *P* < 0.05, *P* < 0.01 and *P* < 0.001, respectively

Pseudo-germ-free mouse model was adopted to confirmed the potential effect of *P. stercorea* interacting with alfalfa in feed on IMF deposition. The AS group displayed significantly higher weights of subcutaneous and epididymal adipose tissues compared to the CON group (*P* < 0.05) (Fig. 6F). Additionally, the fat composition relative to body weight was greater in the AS group than in the AP, CP, and CON groups (*P* < 0.05) (Fig. 6H).

Serum analysis revealed elevated levels of TCHO and LDL-C in the AS group (Fig. 6J, K). Histological examination of mouse subcutaneous adipose tissue using haematoxylin and eosin staining, showed that the mean diameter and area were significantly larger in the AS group than in the other groups (*P* < 0.05) (Fig. 6R, S).

### Micro-CT and immunofluorescence analysis verified the effect of *P. stercorea* on fat deposition

**Fig. 7:**
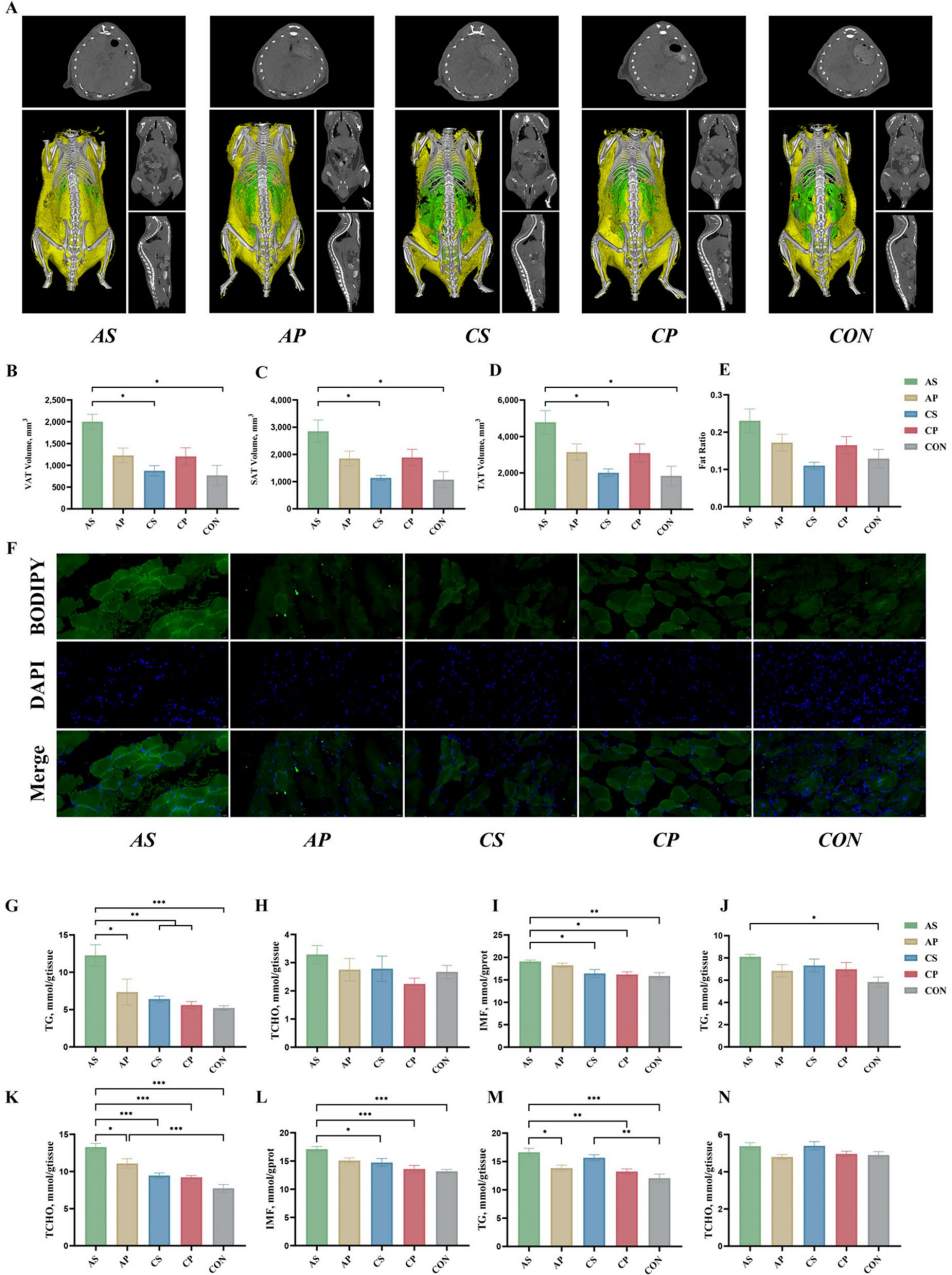
*P. stercorea* increases fat deposition in mouse. **A** In vivo micro-CT scanning of adipose tissue in mice. **B**–**E** Adipose tissue volumes and fat ratio of mouse. F: Representative images of immunofluorescence analysis for Bodipy (green) and 4ʹ,6-diamidino-2-phenylindole (DAPI; blue) as staining on the tibialis anterior muscle sections. **G**–**I** The TG, TCHO and IMF content of mouse tibialis anterior muscle. **J**–**L** The TG, TCHO and IMF content of mouse gastrocnemius muscle. **M**–**N** The TG and TCHO content of mouse liver. *, ** and *** indicate *P* < 0.05, *P* < 0.01 and *P* < 0.001, respectively

High-contrast in vivo micro-CT imaging was performed to identify and quantify fat volumes in different body compartments. The AS group exhibited significantly higher volumes of visceral fat, subcutaneous fat, and total fat compared to the CS and CON groups (*P* < 0.05), while differences in body fat percentage were not significant (Fig. 7A–E), suggesting that *P. stercorea* may promote fat deposition in mice under the conditions of feeding fibre diets. Immunofluorescence images of mouse tibialis anterior muscle showed a similar trend, with the AS group presenting the highest fluorescence intensity (Fig. 7F). The IMF levels in both the tibialis anterior and gastrocnemius muscles were significantly greater in the AS group compared to the other groups (*P* < 0.05) (Fig. 7I, L). In addition, TG content in the tibialis anterior muscle, gastrocnemius muscle and liver, as well as TCHO content in the gastrocnemius muscle, were elevated in the AS group *(P* < 0.05) (Fig. 7G, J, M, K).

### The assessment of glucose homeostasis and pancreatic development

Both *P. stercorea* and *P. copri* were found to increase pancreatic mass and the number of islets, indicating positive effects on pancreatic development in mice. The GTT and ITT revealed that glucose intolerance and insulin resistance were improved by *P. stercorea* under fibre feeding conditions (Fig. S5D–G). The AUC of plasma glucose from GTT was significantly higher in the AS group compared to the other groups (*P* < 0.05).

#### Differences in the structure of the colonic microbiota in mice

To investigate the impact of *P. stercorea* and fibre crosstalk on the colonic microbial communities of mice, we employed 16S rRNA sequencing. PCoA plots based on Bray–Curtis distance revealed differences in microbiota among five groups, and Anosim analysis further confirmed significant differences (R = 0.565, *P* = 0.001) (Fig. S6E). Further groups were combined by feed type and microbial strain and we observed significant differences in microbiota between feeds (R = 0.552, *P* = 0.001) and non-significant differences between strains (R = 0.023, *P* = 0.244) (Fig. S6F, G). PERMANOVA was performed to calculate the contribution of feed type and gavage strains to the microbial communities. The analysis revealed that former explained 24.0% of the variability, while the strain accounted for 10.4% (Fig. S6H). These findings indicate that dietary fibre is the predominant factor influencing gut microbiota.

#### Altered microbial communities in mice driven by *P. stercorea*

**Fig. 8:**
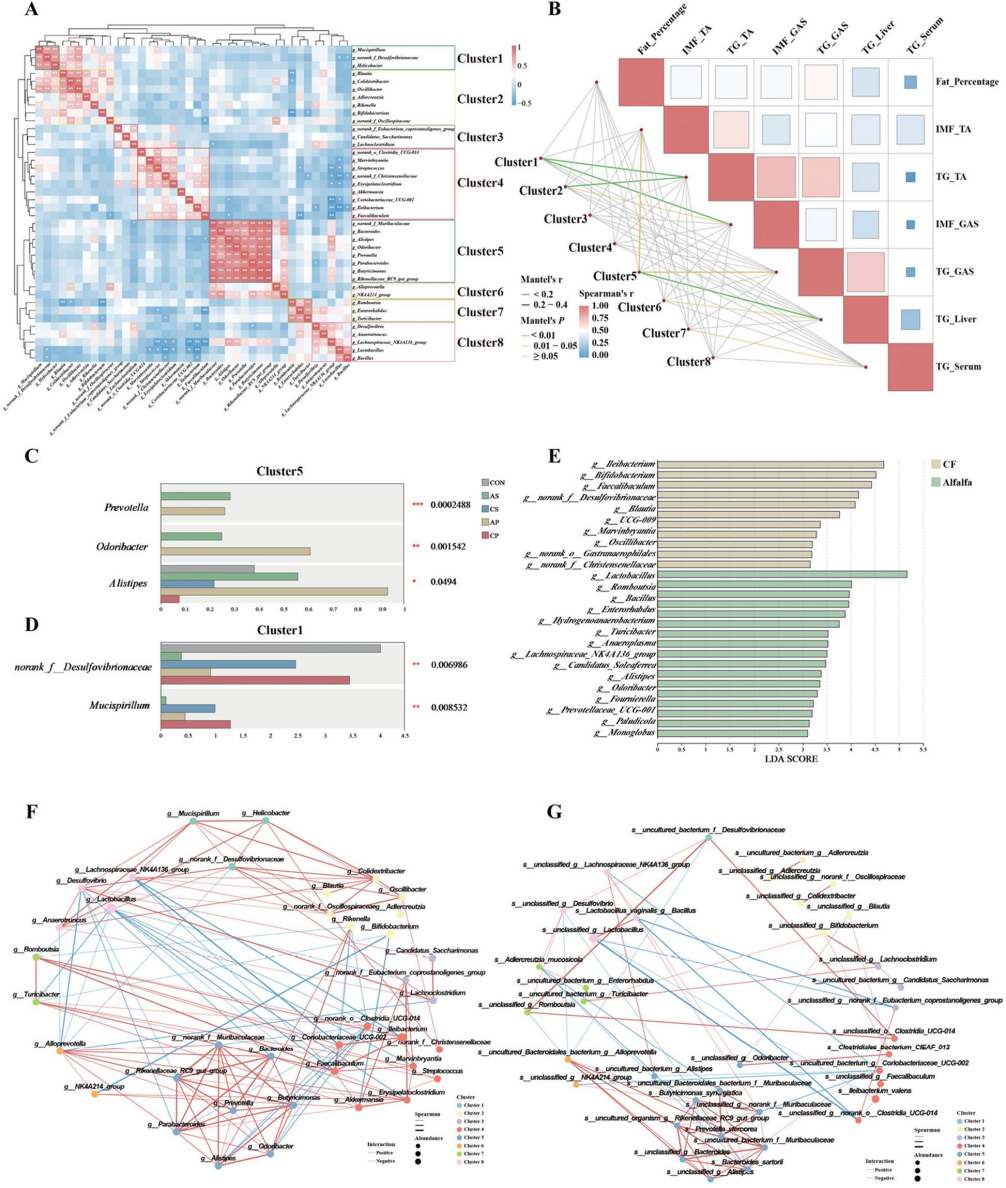
Altered microbial communities in the mouse colon. **A** Heatmap of top 40 genera by Pearson’s rank, the most eight dominant clusters identified are highlighted by different colored boxes. * and ** indicate *P* < 0.05 and *P* < 0.01, respectively. **B** Mantel test of the eight dominant clusters and fat deposition traits, using Spearman's rank correlation coefficient. **C**, **D** Bar charts show differences in relative abundance of the top 40 genera cross groups, Kruskal–Wallis H test used to evaluate significant differences. **E** Histograms of LDA scores reveal the most differentially abundant taxa among different feed types. **F**–**G** Microbial correlation network diagram with Spearman’s rank correlation coefficients. Positive associations are denoted by red lines and negative associations by blue lines

Gut microorganisms form highly dynamic ecosystems shaped by factors such as diet, environment, and host conditions, and typically form complex communities to adapt to changing environments. To clarify how interactions between *P. stercorea* and fibre affect the microbial community in the mouse colon, we performed a co-correlation analysis of the 40 most abundant genera. These genera were organized into eight clusters based on Pearson's rank correlation coefficient, illustrated in the dendrogram on the left side of heat map (marked by the red dashed line), and the goodness of clustering was further confirmed by “clusGap” function of “cluster” R package (Fig. S4C, D). Among these clusters, *Prevotella* and seven other fibre-degrading genera formed cluster 5 (Fig. 8A). Further, we assessed Mantel test between the eight clusters and fat deposition, finding that cluster 5 had the strongest positive correlation with fat deposition, while clusters 1 and 2 had strong negative correlations (Fig. 8B).

The Kruskal–Wallis rank sum test revealed that *Prevotella*, *Odoribacter*, and *Alistipes* in cluster 5 were significantly more abundant in fibre-fed groups (*P* < 0.05). In cluster 1, *norank_f_Desulfovibrionaceae* and *Mucispirillum* showed significantly lower abundance under fibre feeding (*P* < 0.01) (Fig. 8C, D). These results indicate a positive association between Cluster 5, represented by *Prevotella*, and fat deposition, with fibre feeding increasing the abundance of this cluster. Therefore, we combined the samples by feed type and performed LEfSe analysis and found that *Prevotella*, *Odoribacter*, and *Alistipes* were significantly enriched in fibre-rich diets (Fig. 8E).

Correlation networks also showed that the genera in cluster 5 formed a rich and complex network, with *Prevotella* at the centre (Fig. 8F). To investigate the interactions between fibre and *P. stercorea* in more detail, we constructed a species-level correlation network for the AS group, and identify positive associations between *P. stercorea* and *s_Bacteroides sartorii, s_unclassified_g_norank_f_Muribaculaceae, s_uncultured_Bacteroidales_bacterium_f_Muribaculaceae,* and *s_Butyricimonas synergistica* (Fig. 8G). Notably, these associations were absent in the correlation network of the CS group, highlighting the strong influence of dietary fibre in mediating these interactions (Fig. S7B).

#### Analysis of metabolomic differences in colon contents and association with gut microbiome

**Fig. 9:**
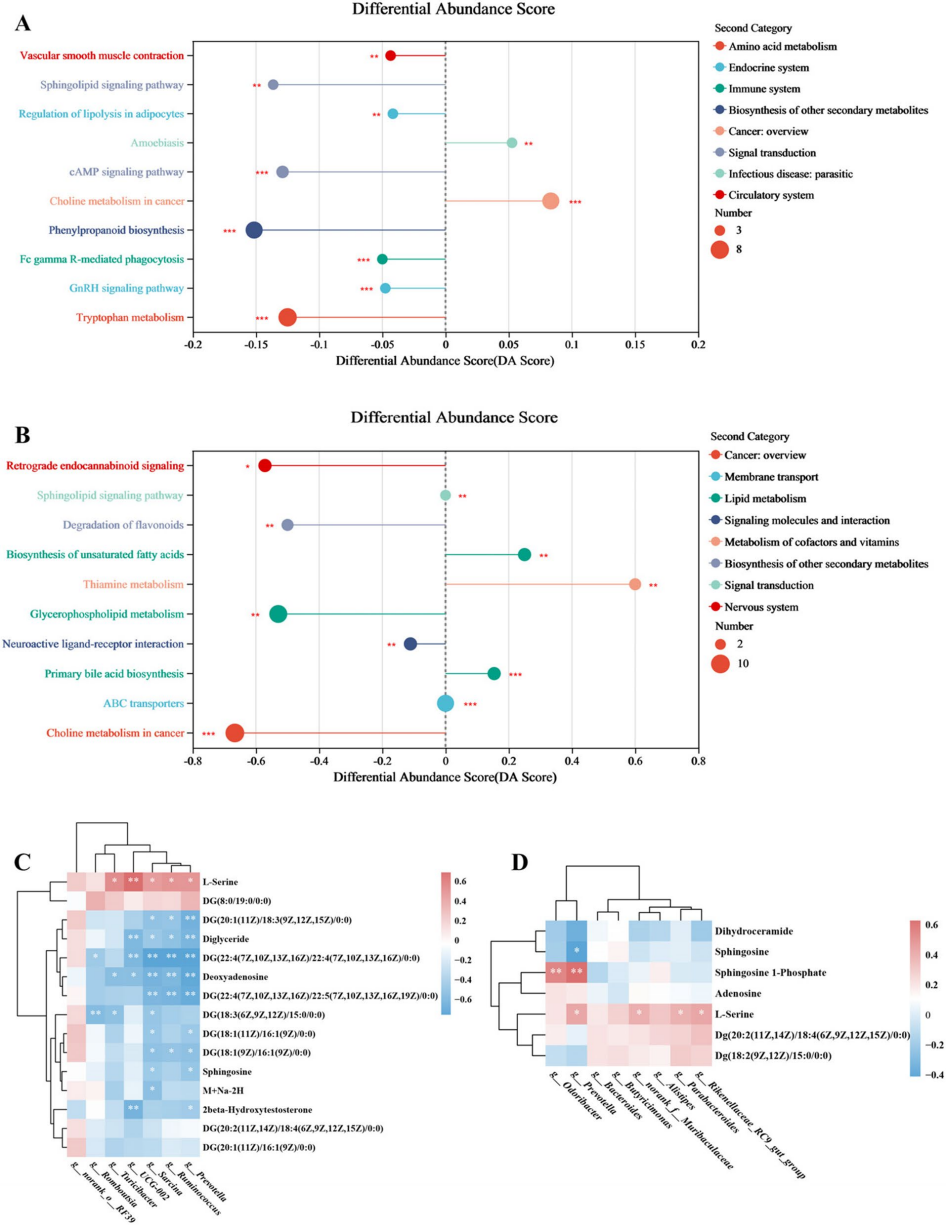
KEGG pathway enrichment and association analysis with gut microbes. **A**–**B** KEGG pathway enrichment of differential metabolites on colon content of pig (**A**) and mice samples (**B**). **C** Spearman association analysis between pig metabolites within Sphingolipid signaling pathway and genera of Cluster2. **D** Spearman association analysis between mice metabolites within Sphingolipid signaling pathway and genera of Cluster5. *, ** and *** indicate *P* < 0.05, *P* < 0.01 and *P* < 0.001, respectively

To assess metabolite differences, we performed LC–MS/MS analysis on colon content samples from both pigs and mice. KEGG pathway enrichment analysis was performed on the differential metabolites to explore the potential metabolic pathway alterations. Sphingolipid signaling pathway and Regulation of lipolysis in adipocytes pathway were significantly down-regulated in JH of 180 d. Biosynthesis of unsaturated fatty acids and Primary bile acid biosynthesis were significantly up-regulated, while Glycerophospholipid metabolism was significantly down-regulated in mice gavaged with *P. stercorea*. Notably, sphingolipid signaling pathway was observed to be significantly different in both pig and mouse metabolomic (Fig. 9A, B).

Next, we performed Spearman correlation analyses between metabolites clustered to sphingolipid signaling pathway and genera related to fat deposition (Cluster2 of porcine microbiome and Cluster5 for mouse microbiome). In the pig samples, we found a significant negative correlation between diglycerides and *Prevotella*, while L-Serine was positively correlated with *Prevotella* in both the porcine and murine samples. In mouse samples, the analysis revealed that sphingosine 1-phosphate had the strongest positive correlation with *Prevotella*, suggesting a potential link between this metabolite and *Prevotella*-driven fat deposition (Fig. 9C, D).

## Discussion

In this study, we utilized a cohort of pigs housed under the same conditions at three points in time, from weaning to slaughter, to analyze in detail the changes in pork quality, lipid metabolic and gut microbiota throughout the entire lifecycle of pigs, with a focus on the potential microbial biomarker associated with IMF deposition. Our analysis revealed that age was the primary factor influencing bacterial community composition, followed by pig breed. Interestingly, *Prevotella* was found to respond to both age and breed factors in the MaAsLin2 analysis.

As a widespread and abundant genus in mammals, *Prevotella* is known to play a crucial role in fibre degradation, producing metabolites like acetate and succinate, and its abundance is closely related to host body mass index, glucose metabolism, and gut health [14, 24]. It is also a core dominant gut microbes of pigs [33, 34]. Previous studies have also shown that *Prevotella*-driven enterotypes are associated with improved growth traits, such as feed intake, weight gain, and reduced diarrhea rates in pigs [35].

In our experiments, *Prevotella* was significantly enriched in JH, and random forest model further confirmed its potential as a microbial biomarker for high IMF deposition. Notably, Cluster 2, centered around *Prevotella*, formed a complex microbial network in which *Prevotella*, *Sarcina*, and *Ruminococcu*s were positively correlated with IMF, marbling, and TG. Among these, *Prevotella* exhibited the strongest correlation with IMF, highlighting its potential role in regulating lipid metabolism and IMF accumulation in the host through interactions within the microbial community.

The human gut microbiota plays a crucial role in the regulation of host metabolism, particularly in relation to dietary intake and fat deposition. Among the diverse microbial populations that inhabit the gut of mammals, *Prevotella* is a prominent genus linked to fibre-rich diets [14]. Members of the *Prevotella* genus are renowned for their capacity to degrade complex polysaccharides and plant fibres. However, *Prevotella* is a highly diverse genus, with a broad range of strains that occupy different ecological niches and exhibit distinct functional capabilities. This functional diversity among *Prevotella* strains is particularly evident in the differences in their unique genes encoding carbohydrate enzymes and polysaccharide utilization sites [14, 21].

*P. copri* is a common inhabitant of the human gut microbiome, exhibiting substantial inter-individual variability. It is divided into four distinct clades, forming the *P. copri* complex [36]. Dietary patterns seem to play a key role in the selection of specific *P. copri* strains. For example, *P. copri* strains from individuals in westernized populations are more efficient at the degradation of complex polysaccharides, metagenomic analysis reported the enrichment in genes encoding enzymes degrade starch, xylans and polygalacturonans. In contrast, strains from Western populations tend to be more enriched in protease genes, reflecting the higher protein content in diets [24].

*P. stercorea* is likely the second most abundant species in the human gut microbiome, following *P. copri*, based on metagenomic data from healthy human gut samples [37]. While *P. copri* is known for its ability to degrade complex hemicellulose, *P. stercorea* appears to have a distinct role in carbohydrate digestion due to its lack of hemicellulolytic gene families. *P. stercorea* contains carbohydrate esterase families that likely function to remove ester modifications from dietary carbohydrates. This process may facilitate the breakdown of these carbohydrates by other gut microbes. Additionally, *P. stercorea* strains also possess sialidases, enzymes that could further assist in the digestion of complex carbohydrates in the gut [25, 37]. The study of Gambian infant’s cohort found, *Prevotella* should not be seen as a monolithic group, the trophic network centered around *P. stercorea* appeared diverse and self-contained and lost parallel with *P. copri*.

In this study, we identified the *P. stercorea *DSM 18206 strain in pig samples and administered to pseudo sterile mouse to examine the impact on IMF deposition, as well as the interaction with dietary fibre. Additionally, group gavaged with *P. copri* was included, which previously linked to fat accumulation in both pigs and mice [26], to further evaluation of effects on IMF deposition.

Our findings demonstrate that *P. stercorea* significantly influences fat accumulation in mouse, particularly when combined with an alfalfa-enriched diet. The gavage of *P. stercorea* on an alfalfa-enriched diet (AS group) led to a notable increase in adiposity, with significantly higher weights of subcutaneous and epididymal adipose tissues compared to the control group. This was accompanied by an increase in adipocyte diameters. The elevated TG and IMF contents in TA and GAS muscles further indicate that *P. stercorea* not only influences adipose tissue but also affects fat deposition in muscle tissue, which could have implications for overall metabolic health. Interestingly, the in vivo micro-CT imaging provided a comprehensive assessment of fat distribution, revealing that both visceral and subcutaneous fat volumes were significantly higher in the AS group, with the body fat percentage remained unchanged, suggesting that *P. stercorea* may specifically enhance fat deposition without altering overall body composition. However, given the differences in fatty acid synthesis and fat deposition between pigs, mice and humans, the applicability of our conclusions to pigs remains uncertain. Therefore, further research using pig models is essential to validate the fat deposition-promoting effect of *P. stercorea* in swine and to explore its potential implications for livestock production and human metabolic health.

Metabolomic analysis further confirmed the potential regulation of fat deposition by *P. stercorea*. In our study, the sphingolipid signaling pathway was consistently altered in both porcine and murine models, suggesting a potential mechanistic link between microbiome and fat deposition. Notably, correlation analysis further revealed that sphingosine 1-phosphate, a bioactive sphingolipid metabolite, was most positively correlated with *Prevotella* [38]. Sphingosine 1-phosphate is known to play a key role in various physiological and pathological processes, including fundamental cellular functions, insulin signaling, and the development of conditions such as diabetes and obesity [39–41]. Studies has shown plasma sphingosine 1-phosphate was specifically associated with fat mass and visceral fat area in type 2 diabetes mellitus [38]. These findings highlight the ability of *P. stercorea* to influence host fat metabolism, particularly in relation to the sphingolipid signaling pathway.

Moreover, the study revealed the beneficial effects of *P. stercorea* combined fibre feeding on glucose metabolism evidenced by the GTT and ITT results, highlighted the effect of dietary fibre on bacterial function and host metabolism. Fibre has been shown to increase host glucose metabolism, mice with resistant dextrin supplementation were dominated by Prevotellaceae, and observed an improvement of insulin resistance [42]. These results highlight the potential role of gut microbes, specifically *P. stercorea*, in modulating lipid metabolic, with implications for understanding how diet and the microbiome interact to affect host metabolism.

Microorganisms naturally form complex and dynamic communities to adapt to changeable environmental conditions [43]. Recent research has highlighted interactions of microbial community members, including cooperative, competitive, or neutral relationships, all of which are vital for maintaining community stability and influencing microbial composition [43–47]. Considering the intricate and social nature of microorganisms [46], it would be one-sided and incomplete to explore the effect of a single strain on host metabolism in isolation from the microbial community. Therefore, 16S rRNA sequencing was used to examine the microbial communities in the colons of mice, providing a more holistic view of the interactions between *Prevotella* species, fibre, and host metabolism.

A co-correlation analysis in the mouse gut revealed *Prevotella* and other fibre-degrading microbes formed a distinct cluster positively correlated with IMF deposition, which was aligned with the previous phenotypes. The abundance of this cluster was significantly higher in fibre-fed groups, highlighting the potential role of fibre in promoting the growth of specific microbial species that influence metabolic outcomes. PERMANOVA further confirmed fibre feeding was the primary factor driving microbiota community structure.

These findings support our hypothesis that *P. stercorea* under dietary fibre can modulate microbial communities, further influence the host fat deposition. The positive associations between *P. stercorea* and other gut microbes further suggest that microbial interactions play a critical role in shaping metabolic outcomes, particularly in response to dietary fibre.

## Conclusions

This study examined the alterations in fat deposition and gut microbes throughout the pig life cycle and identified a potentially functional strain, *P. stercorea*, a biomarker responded to high IMF deposition. The influence of *P. stercorea* on the gut microbiota and its core role in promoting fat accumulation was validated in mice. These findings provide a theoretical basis for developing probiotic strategies aimed at improving pork quality and offer a reference point for the influence of gut microbes in human lipid metabolism.

## Supplementary Information

Additional file 1: Fig. S1. The TG content, enzyme activity and expression of genes related to lipid metabolism. Fig. S2. The α diversity and β diversity of colon microbial diversity. Fig. S3. Multivariable statistical analysis from single time points datasets and the mixed-effects linear regression identified genera associated with age. Fig. S4. Gap Statistic identified the number of clusters for top genera co-correlation analysis. Fig. S5. *P. stercorea* improves glucose intolerance and insulin resistance in mice. Fig. S6. Structural differences in the microbial communities of the mouse colon. Fig. S7. Microbial correlation network analysis and LEfSe analysis of mice.

Additional file 2: Tabel S1. Meat quality and carcass index. Tabel S2. The genera of MaAsLin2 model responding to pig breeds in day 60. Tabel S3. The genera of MaAsLin2 model responding to pig breeds in day 90. Tabel S4. The genera of MaAsLin2 model responding to pig breeds in day 180. Tabel S5. The genera of MaAsLin2 model responding to feed types in day 60. Tabel S6. The genera of MaAsLin2 model responding to feed types in day 90. Tabel S7. The genera of MaAsLin2 model responding to feed types in day 180. Tabel S8. Mantal test. Tabel S9. ASVs belong to *Prevotella* in pig samples. Tabel S10. The sequences of ASVs belong to *Prevotella*.

## Data Availability

The raw sequencing reads of colon intestinal microbes in pigs and mouse were deposited into the NCBI Sequence Read Archive (SRA) database (Accession Number: PRJNA1199807, PRJNA1200223).

## Abbreviations

AP: Alfalfa grass diet with *P. copri* gavaged
AS: Alfalfa grass diet with *P. stercorea* gavaged
ASVs: Amplicon sequence variants
AUC: The area under the curve
BF: Backfat thickness
BLAST: The NCBI Basic Local Alignment Search Tool
CON: Control diet with PBS gavaged
CP: Control diet with *P. copri* gavaged
CS: Control diet with *P. stercorea* gavaged
CSS: Cumulative Sum Scaling
CW: Carcass weight
DDF: Duroc × Landrace × Yorkshire pig with Duroc × Landrace × Yorkshire pig feed group
DF: Duroc × Landrace × Yorkshire pig feed
DJF: Duroc × Landrace × Yorkshire pig with Jinhua pig feed group
DLY: Duroc × Landrace × Yorkshire pig
GTT: Glucose tolerance test
HDL: High-density lipoprotein
HE staining: Hematoxylin-eosin staining
IMF: Intramuscular fat
IMP: Inosine monophosphate acid
ITT: Insulin tolerance test
JDF: Jinhua pig with Duroc × Landrace × Yorkshire pig feed group
JF: Jinhua pig feed
JH: Jinhua pig
JJF: Jinhua pig with Jinhua pig feed group
LDL: Low-density lipoprotein
LDM: Longissimus dorsi muscle
LEfSe: Linear discriminant analysis Effect Size
LP: Leptin
LPL: Lipoprotein lipase
MaAsLin2: Microbiome Multivariate Association with Linear Models
micro-CT: Micro-Computed Tomography
*P. copri*: *Prevotella copri *DSM 18205
*P. stercorea*: *Prevotella stercorea *DSM 18206
PCoA: Principal co-ordinates analysis
PERMANOVA: Permutational multivariate analysis of variance
ROC: The receiver operating characteristic curve
TChol: Total cholesterol
TG: Triglyceride
TSS: Total Sum Scaling
