# Supplementary material for: Prevotella stercorea increases fat deposition in Jinhua pigs fed alfalfa grass-based diets
Source: J Anim Sci Biotechnol. 2025 Jun 24;16:88. doi: 10.1186/s40104-025-01217-6 (PMC12186324; doi:10.1186/s40104-025-01217-6)
Supplement: Supplementary file 1 — Additional file 1: Fig. S1. The TG content, enzyme activity and expression of genes related to lipid metabolism. Fig. S2. The α diversity and β diversity of colon microbial diversity. Fig. S3. Multivariable statistical analysis from single time points datasets and the mixed-effects linear regression identified genera associated with age. Fig. S4. Gap Statistic identified the number of clusters for top genera co-correlation analysis. Fig. S5. P. stercorea improves glucose intolerance and insulin resistance in mice. Fig. S6. Structural differences in the microbial communities of the mouse colon. Fig. S7. Microbial correlation network analysis and LEfSe analysis of mice. [file 40104_2025_1217_MOESM1_ESM.docx]

# **Supplementary Figures**


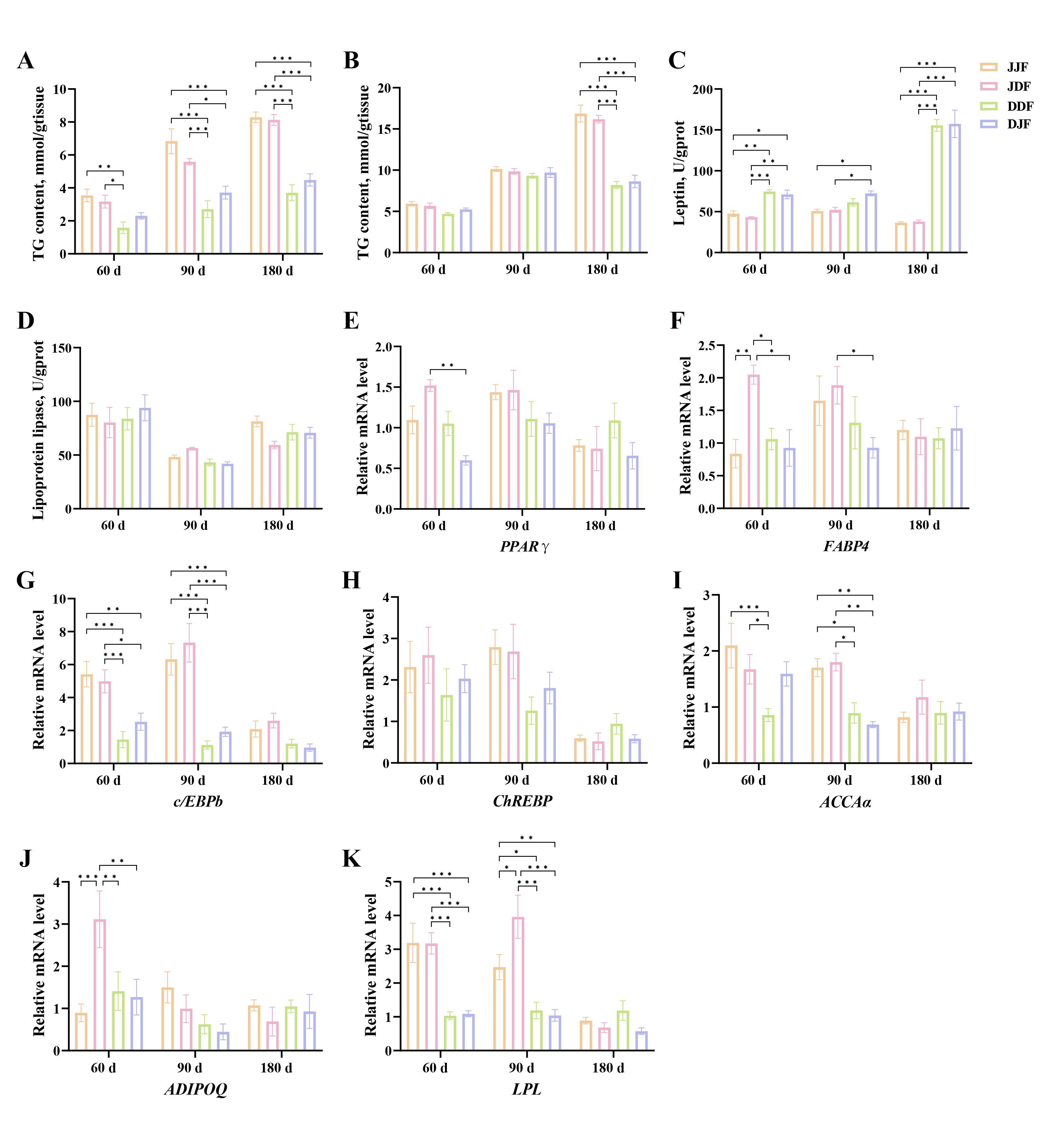


Fig. S1. The TG content, enzyme activity and expression of genes related to lipid metabolism. A: TG content of LDM. B: TG content of liver. C: Leptin enzyme activity. D: Lipoprotein lipase enzyme activity. E-K: Relative mRNA level of genes related to lipid metabolism in LDM. *, ** and *** indicate *P* < 0.05, *P* < 0.01 and *P* < 0.001, respectively.


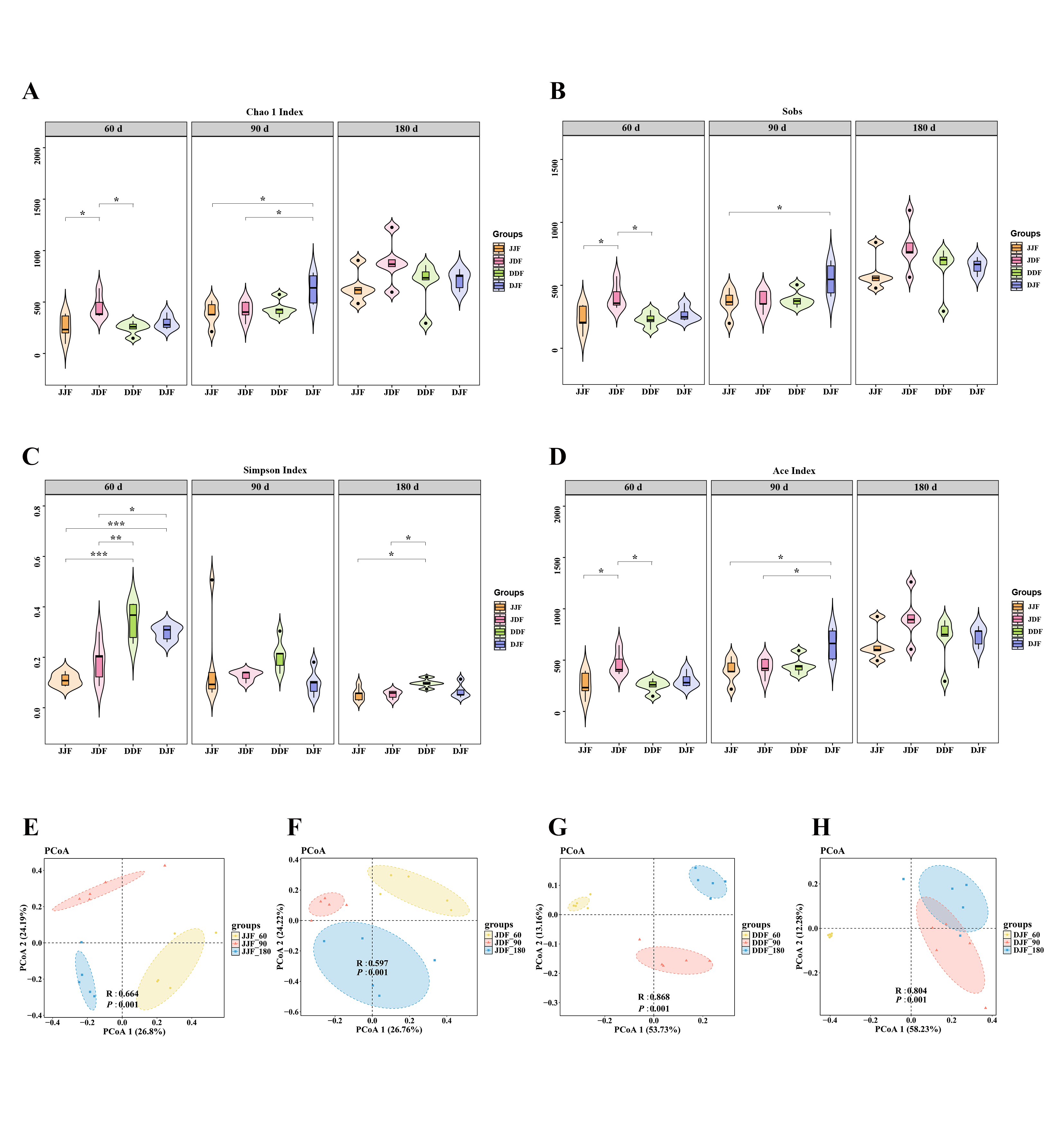


Fig. S2. The α diversity and β diversity of colon microbial diversity. A-D: The α diversity of colonic microbial communities of pigs, Kruskal-Wallis H test is used for multiple comparisons and the significant P value was adjusted by Bonferroni test. Data was statistically significant with a confidence level of 95%. The violin plots showed Chao 1 index (A), Sobs (B), Simpson index (C) and Ace index (D) of different groups among 60 d, 90 d and 180 d. E-H: The β diversity of colon microbial diversity. Principal co-ordinates analysis (PCoA) based on the Bray-Curtis distance of pig colonic microbial communities of JJF (E), JDF (F), DDF (G) and DJF (H) groups. *, ** and *** indicate *P* < 0.05, *P* < 0.01 and *P* < 0.001, respectively.


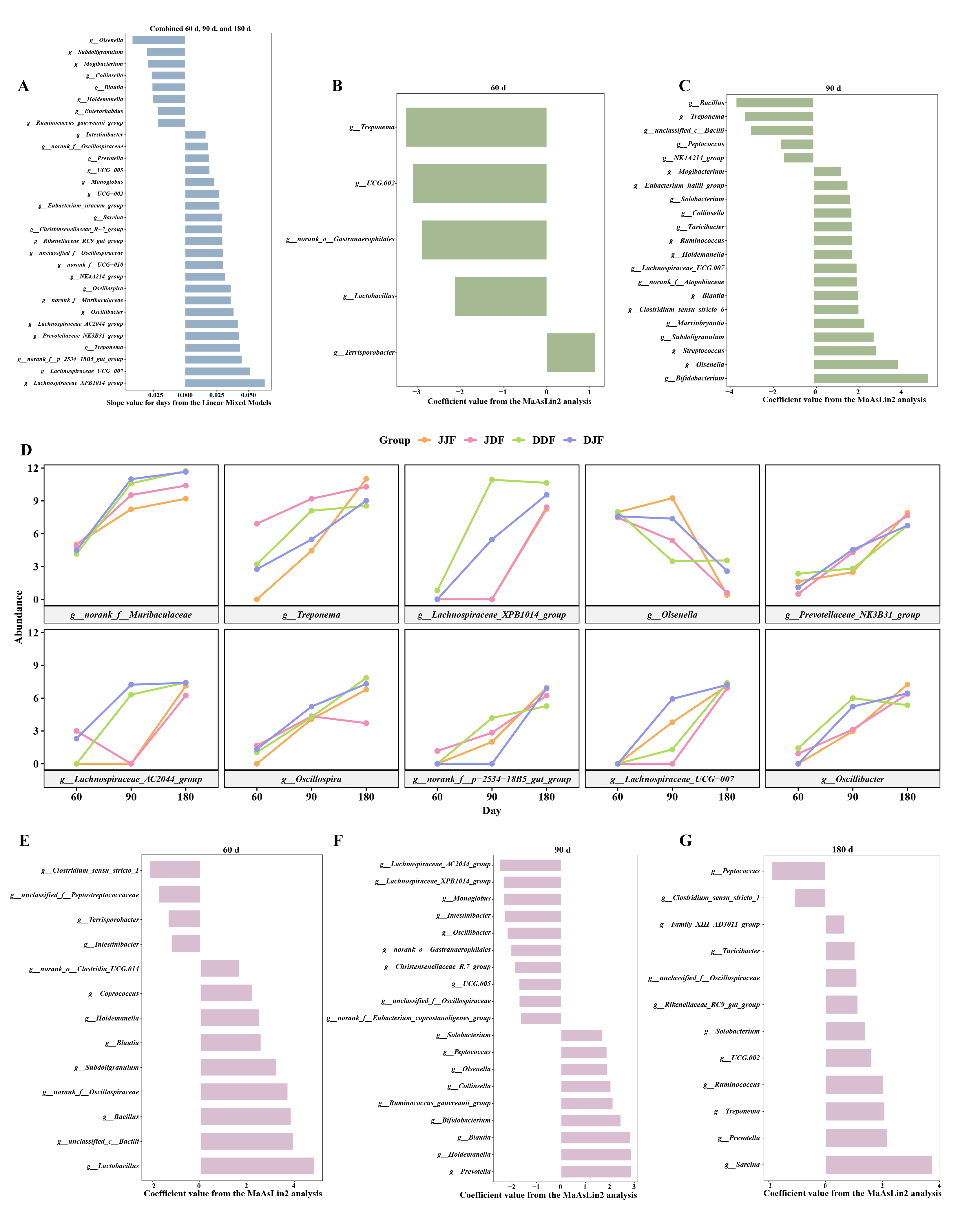


Fig. S3. Multivariable statistical analysis from single time points datasets and the mixed-effects linear regression identified genera associated with age. A: The top 30 slope value of mixed-effects linear regression model in response to age, used TSS transformation followed by CSS normalization, top 60 genera were included while the age, feed and breed were considered as fixed effects, and other detailed parameters of the model were shown in Table 2. B, C: The coefficient values of MaAsLin2 model response to feed type combined 60 d (B) and 90 d (C). D: Lineplot showed increase or decrease in abundance with age for the top ten most strongly age-associated genera by mixed-effects linear regression model. E-G: The coefficient values of MaAsLin2 model used response to pig breed combined 60 d (E), 90 d (F) and 180 d (G).


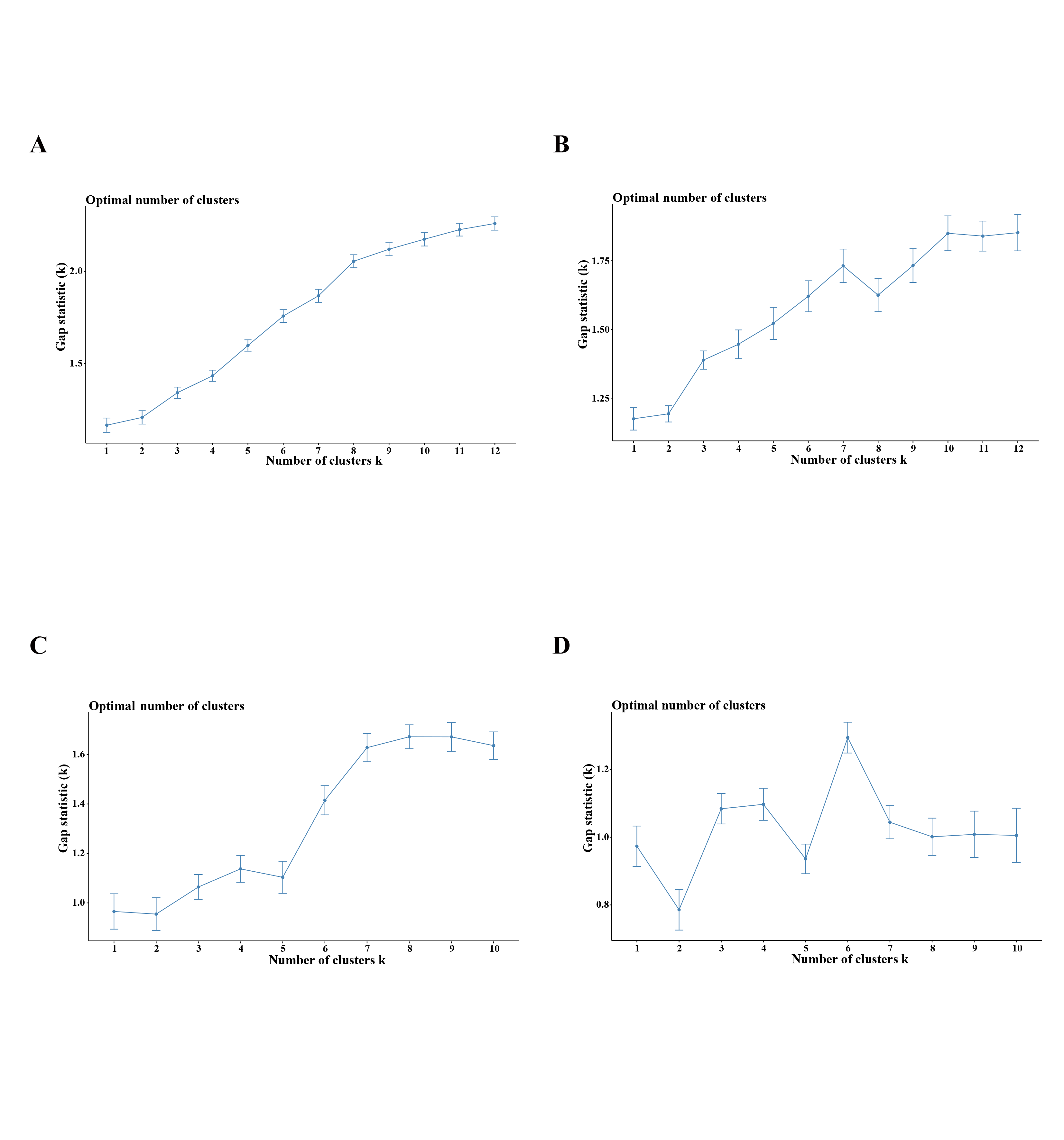


Fig. S4. Gap Statistic identified the number of clusters for top genera co-correlation analysis. The “gap” statistic of clustering was calculated by “clusGap” function from the R package “cluster”, and the bootstrap “B” parameter was set to 100. In pig samples, the “k.max” parameter was set to 12, and the analysis was done with two different “FUNcluster” method including kmeans (A) and cluster:fanny (B). In mice samples, the “k.max” parameter was set to 10, and the analysis was done with two different “FUNcluster” method including kmeans (C) and cluster:fanny (D). Gap statistic values begin to stabilize, represents the optimal number of clusters reached.


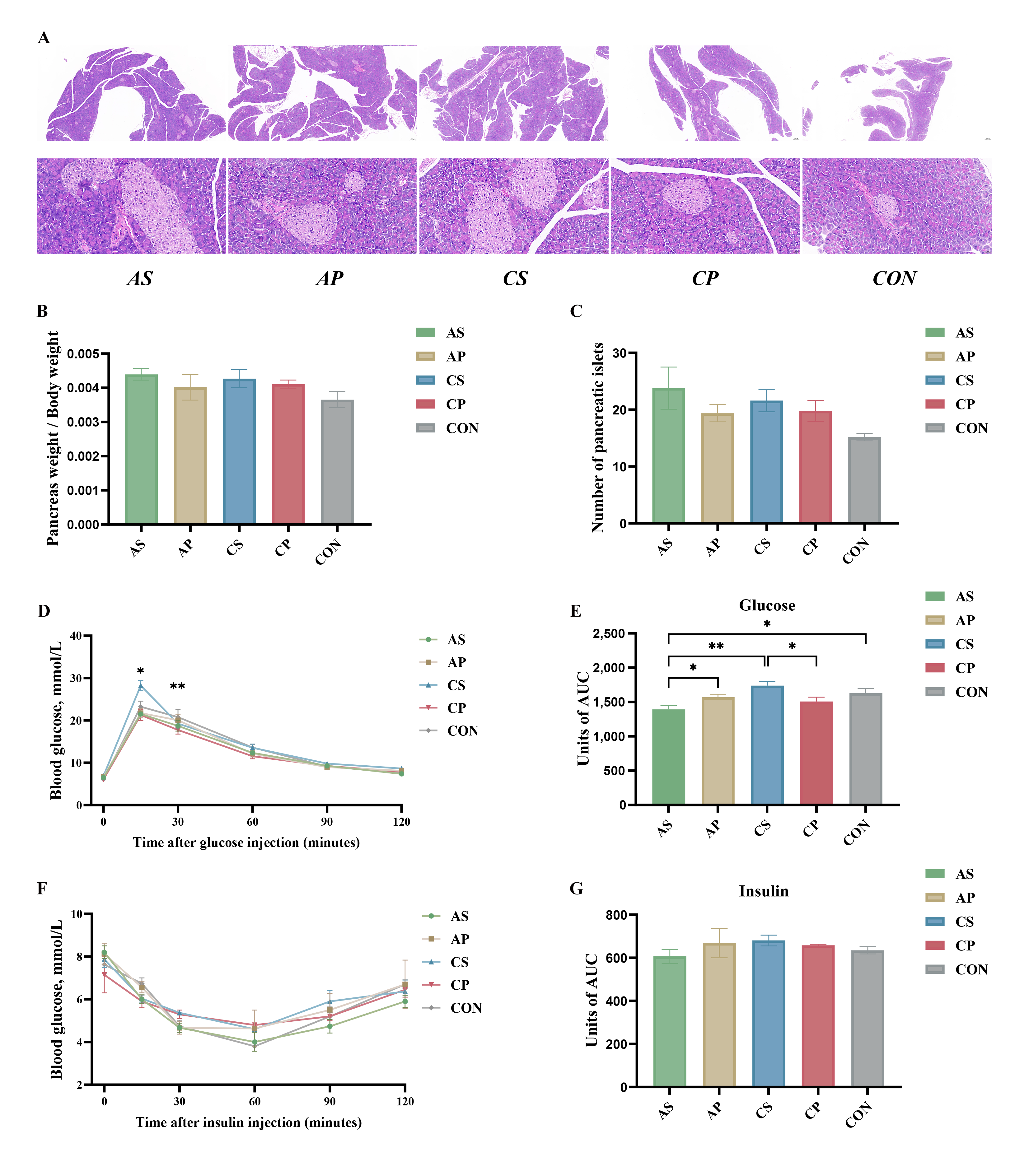


Fig. S5. *P. stercorea* improves glucose intolerance and insulin resistance in mice. A: Histological images depict mouse pancreas tissue using haematoxylin and eosin staining. B: Pancreas / body mass ratio. C: The number of pancreatic islets. D: GTT. E:  The AUC of GTT. F: ITT. G: The AUC of ITT. * and ** indicate *P* < 0.05 and *P* < 0.01 respectively.


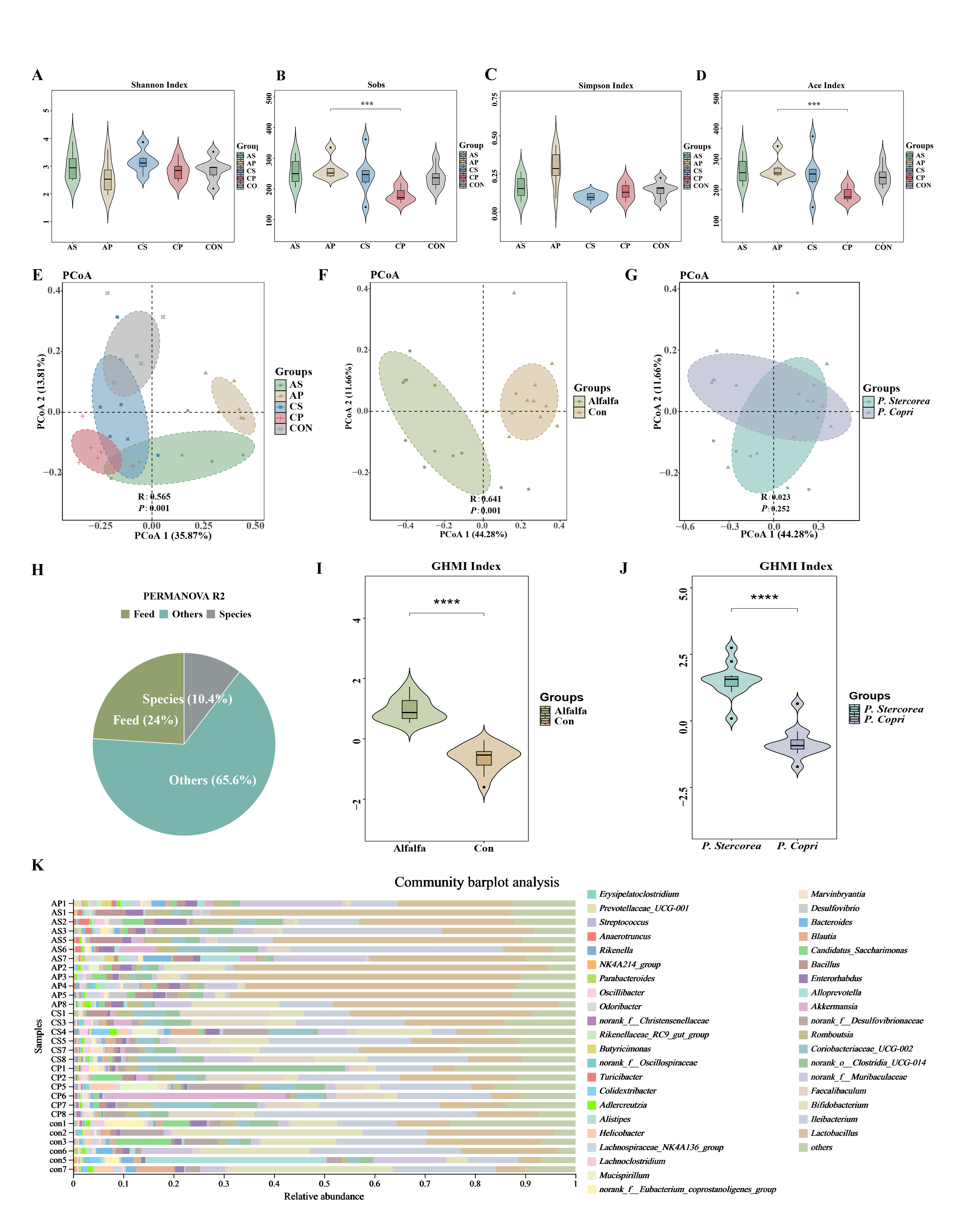


Fig. S6. Structural differences in the microbial communities of the mouse colon. A-D: The α diversity of colonic microbial communities; Data are expressed as minimum to maximum (n = 6). E-G: The β-diversity of colon microbial diversity, PCoA based on the Bray-Curtis distance. H: Contribution of feed type and gavage microbial strains to the colonic microbiota of mice. I-J: Colonic gut microbiome health index (GMHI) in mice. K: Community composition of colonic microbiota of mouse at the genus level.


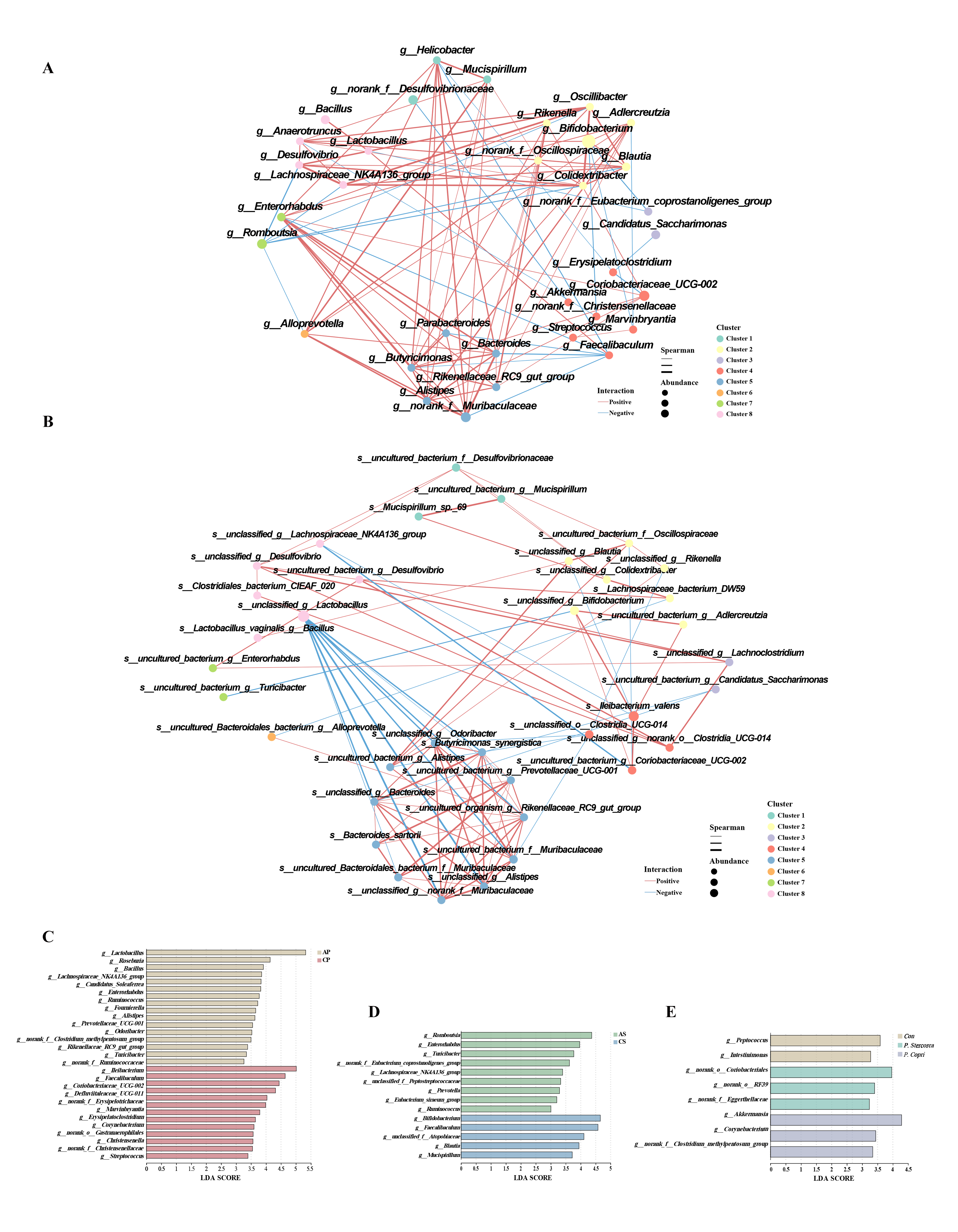


Fig. S7. Microbial correlation network analysis and LEfSe analysis of mice. A, B: Microbial correlation network diagram with Spearman’s rank correlation coefficients. (A): The correlation network on genera level for the control feed group (CS and CP groups). (B): The correlation network on species level for the CS group. Positive associations are denoted by red lines and negative associations by blue lines. C-E: Histograms of LDA scores reveal the most differentially abundant taxa among different feed types (C, D) and different species (E).
